# Pestalotiopols E–J, Six New Polyketide Derivatives from a Marine Derived Fungus *Pestalotiopsis* sp. SWMU-WZ04-1

**DOI:** 10.3390/md22010015

**Published:** 2023-12-26

**Authors:** Liyuan Jiang, Baorui Teng, Mengyu Zhang, Siwei Chen, Dan Zhang, Longfei Zhai, Jiafu Lin, Hui Lei

**Affiliations:** 1 School of Pharmacy, Southwest Medical University, Luzhou 646000, China; jiangly06@163.com (L.J.); tengbaorui01@163.com (B.T.); myzhang65@163.com (M.Z.); chensiwei2021@swmu.edu.cn (S.C.); zhangdan@swmu.edu.cn (D.Z.); 2 Antibiotics Research and Re-Evaluation Key Laboratory of Sichuan Province, Sichuan Industrial Institute of Antibiotics, Chengdu University, Chengdu 610106, China; z960306@163.com; 3 School of Pharmacy, Chengdu University, Chengdu 610106, China

**Keywords:** *Pestalotiopsis* sp., polyketide, sponge-derived fungus, antibacterial activity, cytotoxicity

## Abstract

Chemical epigenetic cultivation of the sponge-derived fungus *Pestalotiopsis* sp. SWMU-WZ04-1 contributed to the identification of twelve polyketide derivatives, including six new pestalotiopols E–J (**1**–**6**) and six known analogues (**7**–**12**). Their gross structures were deduced from 1D/2D NMR and HRESIMS spectroscopic data, and their absolute configurations were further established by circular dichroism (CD) Cotton effects and the modified Mosher’s method. In the bioassay, the cytotoxic and antibacterial activities of all compounds were evaluated. Chlorinated benzophenone derivatives **7** and **8** exhibited inhibitory effects on *Staphylococcus aureus* and *Bacillus subtilis*, with MIC values varying from 3.0 to 50 μg/mL. In addition, these two compounds were cytotoxic to four types of human cancer cells, with IC_50_ values of 16.2~83.6 μM. The result showed that compound **7** had the probability of being developed into a lead drug with antibacterial ability.

## 1. Introduction

Marine fungi provide a prospective source for the discovery of novel drug leads [1]. Traditional methods used in the investigation of marine fungi mainly focus on sample collection, the cultivation of fermentation broth, and mycelium. However, it is becoming difficult to discover novel structurally and biologically active metabolites using these traditional methods, but the development of genetic technology can assist [2]. Research findings demonstrate that a large portion of gene clusters are silenced under standard fermentation conditions [3,4,5]. To explore these potential secondary metabolites, activation of silent biosynthetic gene clusters has become an important strategy. At present, the silent gene clusters have been activated through multiple strategies, such as the OSMAC strategy [6,7,8,9,10], epigenetic modification [6,11,12], and genome mining. Of these, the chemical epigenetic strategy has proved to be an effective strategy for enhancing cryptic secondary metabolites [13].

**Figure 1:**
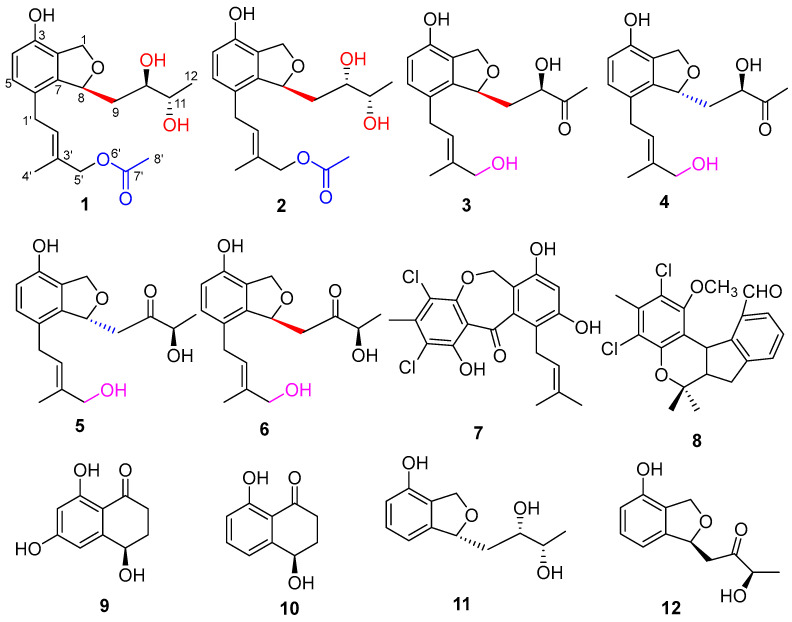
Chemical structures of compounds **1**–**12**.

During our ongoing efforts to discover more new and biologically active secondary metabolites from sponge-derived fungi, we applied the “epigenetic modification” strategy to the investigation of *Pestalotiopsis* sp., including 5-aza-2-deoxycytidine and suberoylanilide hydroxamic acid (SAHA). During this study, six new polyketide derivatives, pestalotiopols E–J (**1**–**6**), and six known analogues (**7**–**12**) (Figure 1), pestalachloride E (**7**) [14], (±)-pestalachloride D (**8**) [15], 3,4-dihydro-4, 6, 8-trihydroxy-1(2H)-naphthalenone (**9**) [16], isosclerone (**10**) [17], isobenzofuranyl derivative (**11**) [18], and (R)-3-hydroxy-1-[(S)-4-hydroxy-1,3-dihydroisobenzo-furan 1-yl] butan-2-one (**12**) [19], were obtained from sponge-derived fungi *Pestalotiopsis* sp. ^1^H and ^13^C assignments of the pestalotiopols E–J (**1**–**6**) were accomplished, the absolute configurations of the compounds were determined by CD techniques, and furthermore, the biological activities of the separated organic molecules were assessed.

## 2. Results

**Figure 2:**
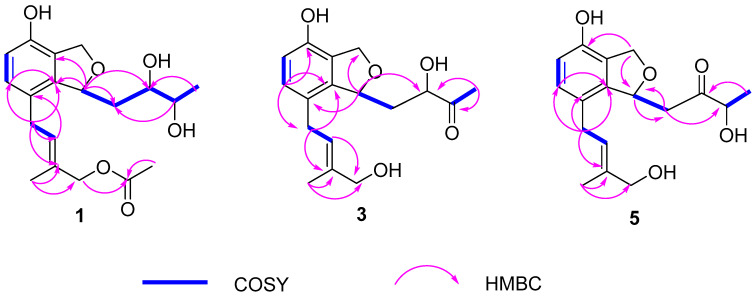
COSY and key HMBC correlations of **1**, **3**, **5**.

Compound **1** appeared as a white solid, and its molecular formula was C_19_H_26_O_6,_ whose quasi-molecular ion peak was located at *m*/*z* 373.1635 [M + Na]^+^ by HRESIMS. The ^1^H NMR spectrum (Table 1) showed two aromatic proton signals [*δ*_H_ 6.60 (d, *J* = 8.1 Hz, H-4), 6.87 (d, *J* = 8.1 Hz, H-5)], which belong to a 1,2,3,4-tetrasubstituent benzene, and one olefinic proton signal [*δ*_H_ 5.57 (brt, *J* = 7.2 Hz, H-2’)]. The ^13^C NMR spectrum of compound **1** showed 19 carbon resonances (Table 1), involving three CH_3_, four CH_2_, three sp^2^-CH carbons, and five not-containing proton carbons. One carbonyl carbon was proposed based on resonances at *δ*_C_ 172.8, and five oxygenated carbon signals at *δ*_C_ 71.0, 71.0, 71.7, 73.3, 82.3. The ^1^H NMR data was initially analyzed, and it was found that **1** had similar structural characteristics to heterocornol L [20]. The major difference was that a carbonyl signal was missing and an oxygenated methine was present in **1**. This was proved by the HMBC correlations between H-10 (*δ*_H_ 3.71) and C-11 (*δ*_C_ 71.7), C-9 (*δ*_C_ 39.1), and C-12 (*δ*_C_ 18.9). The location of the vicinal diol side chain was confirmed by HMBC correlations from H-8 (*δ*_H_ 5.53) to C-7 (*δ*_C_ 143.4.), C-6 (*δ*_C_ 125.9), C-10 (*δ*_C_ 73.3), C-1 (*δ*_C_ 71.0), and ^1^H–^1^H COSY correlations of H-8/H-9/H-10/H-11/H-12 (Figure 2). Furthermore, HMBC correlations found H-1’(*δ*_H_ 3.27) to C-5’ (*δ*_C_ 71.0), C-2’ (*δ*_C_ 128.8), C-3’ (*δ*_C_ 132.3), and C-7 (*δ*_C_ 143.4), together with ^1^H–^1^H COSY correlations located connections from H-1’/H-2’ suggested the existence of 5’-O-acetyl isoamylene group located at C-6 in **1** (Figure 2). Therefore, the planar structure of **1** was established.

**Figure 3:**
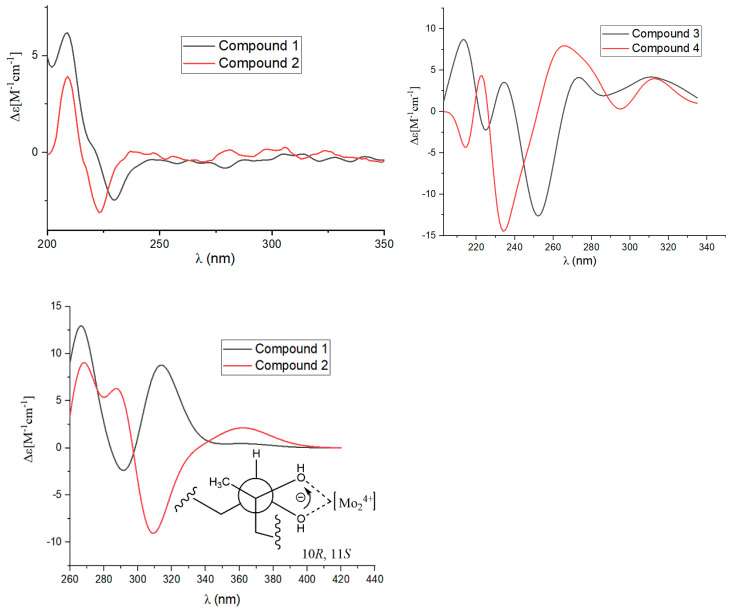
CD spectra of **1**–**4** as well as Mo_2_(AcO)_4_-induced CD spectra **1**–**2**.

To establish its relative configuration, the NOESY spectrum of **1** was performed; unfortunately, the NOESY spectrum of **1** was not useful for the assignment of the relative configuration at C-10 and C-11. According to the previously reported [21,22,23,24], in *rythron* vicinal diols (*J* > 4.0 Hz), the value of the coupling constant of the methine hydrogens was larger than 4 Hz, while in *threo* ones (*J* < 2.0 Hz), it is smaller than 2 Hz. On this account, consistent with the known pyriculin A and B [25] (*J* = 3.7/3.8 Hz), heterocornol I [20], indicating that the configurations of C-10 and C-11 in **1** (*J* = 4.7 Hz) were suggested as *erythro*. The absolute configuration of 1 (8*S*,10*R*,11*S*) was elucidated from the observed positive Cotton effect at 210 nm and positive Cotton effect at 313 nm in the CD and Mo_2_(AcO)_4_-induced CD spectra of **1**, respectively (Figure 3) [19].

Compound **2** was purified as a white solid with the molecular formula C_19_H_26_O_6_, determined from the HRESIMS data. The NMR data of **2** were very similar to those of **1**, which manifests that both of them had the same planar structures. Compared with compound **1**, the configuration at C-10 and C-11 in the vicinal diol side chain was discrepant, which was proved by the chemical shifts of C-8 (Δ*δ*_C_ 1.1 ppm), C-9 (Δ*δ*_C_ −1.8 ppm), C-10 (Δ*δ*_C_ 1.1 ppm), and C-11 (Δ*δ*_C_ −1.8 ppm). According to the chemical shift of C-10 (*δ*_C_ 74.4) and C-11 (*δ*_C_ 69.9) [26], and the coupling constant between H-10 and H-11 (1.6 Hz), the configuration of C-10 and C-11 was inferred to be *threo*. We speculated that **2** had the same configuration at C-8 (8*S*) as **1** according to their closely similar CD spectrum. The absolute configuration of C-10,11-diols in **2** was determined by Mo_2_(AcO)_4_-induced CD (Figure 3).

In order to further confirm the absolute configuration of **2**, the modified Mosher’s method was performed (**2a** and **2b**) [27,28,29,30] (Figures S36 and S37). Treatment of **2** with [(*S*)-MTPA] and (*R*)-MTPA gave (*S*)-MTPA ester (**2a**) and (*R*)-MTPA ester (**2b**), respectively. The Δ*δ* (*δS*–*δR*) values of **2a** and **2b** established the absolute configuration of C-10 and C-11 in **2**. Thus, compound **2** was assigned and named pestalotiopol F.

Compound **3** appeared as a white solid, and the molecular formula was determined to be C_17_H_22_O_5_. Analyses of the 1D/2D NMR data of **3** suggested that compound **3** was similar to vaccinol H [31]. One additional hydroxymethyl group (*δ*_C_ 61.4) was observed, which was established by HMBC correlations from H-2’ (*δ*_H_ 5.30) to C-7 (*δ*_C_ 142.8), C-1’ (*δ*_C_ 31.2), C-5’ (*δ*_C_ 61.4), and C-6 (*δ*_C_ 126.5), from H-5’ (*δ*_H_ 4.19/4.20) to C-4’ (*δ*_C_ 21.5), C-3’ (*δ*_C_ 136.6), and C-2’ (*δ*_C_ 127.1). In terms of the positive Cotton effect at 212 nm and the biosynthetic pathway, along with the similar NMR data between vaccinol H and **3**, the configuration of **3** was confirmed to be 8*R*, 10*R*. The CD spectrum of (8*R*, 10*R*)-**3** helped reconfirm the configuration of **3** (Figure 3).

Compound **4** is presented as a white solid with a molecular formula of C_17_H_22_O_5_, with its excimer ion peak located at *m*/*z* 307.1532 [M + H]^+^. The NMR data indicated the same planar structure as **3** (Table 2). The differences between them were due to the variations in chemical shifts of C-8 (*δ*_C_ 81.5 in **3** vs. *δ*_C_ 81.2 in **4**) and C-9 (*δ*_C_ 39.7 in **3** vs. *δ*_C_ 44.3 in **4**). These results indicated both compounds differed in the relative configuration at C-8, which was similar to that of vaccinols H and I. After contrasting the similarities in chemical shifts between **3** and **4**, and between vaccinol H and vaccinol I, together with biogenetic considerations, we suggested that the absolute configuration of **4** was the 8*S*, 10*R* configuration. These assignments were further confirmed by the CD spectrum of **4** (Figure 3). Thus, we determined the structure of **4** and named it pestalotiopol H.

Compounds **5** and **6** were acquired as a mixture in a nearly 1:1 ratio, which was established by their HRESIMS and the ^13^C NMR data. The NMR data of **5** and **6** resembled those of **3** and **4**, except for a carbonyl group at C-10 (*δ*_C_ 211.8/211.5) in **5*/*6** and C-11 (*δ*_C_ 213.5/212.7) in **3**/**4**. Compared with **5**, the difference was that the chemical shift of C-8 (*δ*_C_ 79.4), C-9 (*δ*_C_ 38.6), and C-10 (*δ*_C_ 211.5) of **6** shifted to upfield (Δ0.5 ppm, Δ4.5 ppm, Δ0.3 ppm), respectively. The conclusions mentioned above were confirmed by the HMBC correlations between H-8 and C-2/C-7/C-6/C-10, between H-5’ and C-4’/C-3’/C-2’, between H-1’ and C-2’/C-3’/C-5/C-6, and the COSY cross peak of H-1’/H-2’, H-8/H-9, and H-11/H-12. The absolute configurations of C-8 and C-11 in **5** and **6** were assigned 8*R*, 10*R*, and 8*S*, 10*R*, respectively, which were consistent with other analogs from the marine fungus *Pestalotiopsis vaccinii* [31]. Considering that two strains belong to the same genus, *Pestalotiopsis*, we proposed that they arose from the same biosynthetic pathway and shared the chiral center.

**Scheme 1:**
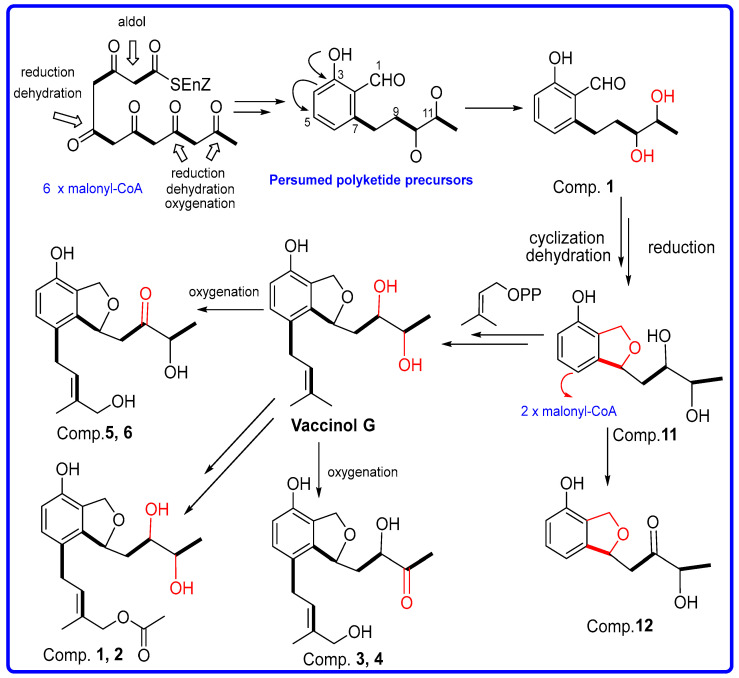
Plausible biosynthetic pathway of compounds **1**–**6**, **11**, **12**.

Biosynthetically, a precursor polyketide substance is commonly produced by microorganisms in the synthesis of aromatics and macrolides. Compounds (**1**–**6**, **11**, **12**) originated from malonyl-CoA by reduction, dehydration, cyclization, and oxidation to form the different polyketide precursors and subsequently formed compounds (**1**–**6**, **11**, **12**) after diverse transformations (Scheme 1).

In the present study, polyketide derivatives **1**–**12** were evaluated for their cytotoxic activities via MTT assay (Table 3). The cytotoxicity results demonstrated that compound **7** was mildly cytotoxic to HepG2, with IC_50_ values of 16.2 μM; the IC_50_ values of **8**, which was weakly cytotoxic to human cancer cells, ranged from 34.8 to 63.1 μM; unfortunately, compounds **1**–**6**, at concentrations of 100 μM, did not show cytotoxicity against these tumor cell lines. Bacteriostatic effects **1**–**12** were performed (Table 3). Compounds **7** and **8** showed antibacterial effects on *Staphylococcus aureus* and *Bacillus subtilis*, with MIC values varying from 3 to 50 mg/mL. No obvious bioactivities were found in the other compounds at 100 μM or 100 mg/mL. The results showed that there was considerable potential to develop compound **7** as a lead drug with antimicrobial activity.

In order to further verify the activity of compounds **7** and **8** against HepG2, the anti-apoptotic protein Bcl-2 (PDB ID: 4LVT) was used as a target for molecular docking. Compounds **7** and **8** bound to ASN9, TRP192, ILE186, TRP141, PHE195, TRP141, LEU198, TYR199, and GLN187 residues and formed hydrogen bonds and non-polar interactions, respectively. Noticeably, the docking results showed that compounds **7** and **8** were well matched in the docking body of 4LVT and had good interaction with 4LVT. The binding free energies of systems were negative (−5.11 kcal/mol and −6.87 kcal/mol, respectively), indicating that the binding of proteins and small molecular ligands was a spontaneous process.

## 3. Materials and Methods

### 3.1. General Experimental Procedures

An MCP500 polarimeter (Anton) was used to record optical rotations in CH_3_OH. IR and HRESIMS were performed on a Shimadzu IR and a Bruker maXis TOF-Q mass spectrometer, respectively. Two different Bruker spectrometers (AVANCE III-400 and AV-600) were used for the NMR data collection, and the NMR data were recorded using TMS as an internal standard, d in ppm rel at 25 °C. Column chromatography involved normal-phase silica gel (100–300 mesh), Sephadex LH-20 (MeOH), and reversed-phase YMC ODS-A (50 μm) being used, while precoated silica gel GF254 plates (0.20–0.25 mm in thickness) were used for thin-layer chromatography (TLC) analyses, and the spots were visualized by UV light (254 nm) and colored by spraying heated silica gel plates with 10% H_2_SO_4_ in ethanol. Preparative HPLC was conducted on a SAIPURUISHE system equipped with a UV detector, an ODS column (YMC-5μm, ODS-A, 250 mm × 10 mm), a flow rate of 2.0 mL/min, and a column temperature of 25 °C. Circular dichroism (CD) spectra were recorded on a Chirascan circular dichroism spectrometer.

### 3.2. Fungal Material

The strain (SWMU-WZ04-1) was obtained from the sponge collected on Weizhou Island, China, and identified as *Pestalotiopsis* sp. SWMU-WZ04-1 by sequence alignment of the 18S rRNA gene. During its initial growth stage on a PDA plate, the mycelium of *Pestalotiopsis* sp. SWMU-WZ04-1 showed a french grey, which gradually transitioned to a brown color. As growth progressed, the mycelium produced conidia along with small oil droplets on the surface.

### 3.3. Fermentation, Extraction, and Isolation

The fungus SWMU-WZ04-1 was cultivated on a rice culture medium (200 g rice, 3% sea salt, 200 mL water, 10 μM of 5-aza-2-deoxycytidine and suberoylanilide hydroxamic acid (SAHA), 120 flasks) for 40 days at a temperature of 28 °C. After fermentation, the fungal culture was exhaustively extracted with EtOAc to obtain a crude extract (48.9 g).

The crude extract was fractionated on a normal-phase column using a stepped gradient elution with petroleum ether/EtOAc (30: 1 to 0: 1, *v*/*v*) to obtain 8 fractions (Fr.1–Fr.8). Fr.3 was applied to a normal-phase column (petroleum ether/EtOAc, 15:1-3:1) to obtain three subfractions (Frs. 3.1–3.3). Fr. 3.2 was purified with Sephadex LH-20 (MeOH) and further purified by HPLC eluting (MeOH/H_2_O 60%) to obtain compounds **3** (8.0 mg) and **4** (3.0 mg). Fr. 3.3 was further separated by an ODS column eluting with MeOH/H_2_O (60%) to obtain subfractions (Fr.3.3.1–Fr.3.3.4). Fr.3.3.2 was purified by Sephadex LH-20 column (MeOH) and HPLC (60%, MeOH/H_2_O) to obtain **11** (6.0 mg) and **12** (5.0 mg). Fr. 4 was subjected to silica gel (petroleum ether-EtOAc, 3:1-0:1) and further separated by (70%, MeOH/H_2_O) HPLC and Sephadex LH-20 (MeOH) to yield **1** (7.0 mg) and **2** (4.0 mg). Fr. 5 was applied by Sephadex LH-20 chromatography (MeOH) and HPLC (60% MeOH/H_2_O) to afford **7** (10.0 mg) and **8** (3.0 mg). Fr. 6 was further divided into seven subfractions (Frs.6.1–6.7) by silica gel cc (CH_2_Cl_2_-Acetone, 6:1-0:1). Frs.6.3 was applied by Sephadex LH-20 (MeOH) and HPLC (H_2_O/MeOH) to yield **5/6** (3.0 mg). Frs. 6.6 was applied by HPLC (MeOH/H_2_O, 45%) to afford **9** (7.0 mg) and **10** (7.0 mg).

*Pestalotiopol E* (**1**): white solid; [α${]}_{D}^{25}$–55 (c 0.3, MeOH); UV (MeOH) *λ*max (log *ε*) 254 (2.24), 275 (3.28) nm; ^1^H NMR and ^13^C NMR data, Table 1; HRESIMS *m/z* 373.1635 [M + Na]^+^ (calcd for C_19_H_26_NaO_6_, 373.1632).

*Pestalotiopol F* (**2**): white solid; [α${]}_{D}^{25}$–50 (c 0.4, MeOH); UV (MeOH) *λ*max (log *ε*) 246 (3.18) nm; ^1^H NMR and ^13^C NMR data, Table 1; HRESIMS *m*/*z* 373.1658 [M + Na]^+^ (calcd for C_19_H_26_NaO_6_, 373.1645).

*Pestalotiopol G* (**3**): white solid; [α${]}_{D}^{25}$−10 (c 0.4, MeOH); UV (MeOH) *λ*max (log *ε*) 254 (2.60), 210 (3.36) nm; ^1^H NMR and ^13^C NMR data, Table 1; HRESIMS *m*/*z* 305.1391 [M − H]^−^ (calcd for C_17_H_21_O_5_, 305.1409).

*Pestalotiopol H* (**4**): white solid; [α${]}_{D}^{25}$+14 (c 0.2, MeOH); UV (MeOH) *λ*max (log *ε*) 246 (3.23), 210 (3.56) nm; ^1^H NMR and ^13^C NMR data, Table 2; HRESIMS *m*/*z* 307.1532 [M + H]^+^ (calcd for C_17_H_23_O_5_, 307.1525).

*Pestalotiopol I and J* (**5/6**): white solid; UV (MeOH) *λ*max (log *ε*) 254 (3.56) nm; ^1^H NMR and ^13^C NMR data, Table 2; HRESIMS *m*/*z* 307.1533 [M + H]^+^ (calcd for C_17_H_23_O_5_, 307.1526).

### 3.4. Mo_2_(AcO)_4_-Induced CD

The Mo_2_(AcO)_4_ solution was prepared with DMSO; subsequently, compounds **1** and **2** were added to the stock solution, respectively. The CD spectra of compounds **1** and **2** were recorded immediately after every 10 min for 30 min to form stationary Mo_2_(AcO)_4_-induced CD spectra [32].

### 3.5. Preparation of (S)- and (R)-MTPA Esters of 2

Each of duplicate **2** (1.7 mg) in 0.3 mL unhydrous pyridine-*d*_6_ in NMR tubes was reacted with (*S*)- and (*R*)-MTPA (160 μL, for **2**), respectively. The reaction was performed at 50 °C for 15 h. Then, the ^1^H NMR data of the (*S*)- and (*R*)-MTPA esters were obtained without purification [33,34,35,36].

### 3.6. Cytotoxicity Assay

The MTT method was applied to analyze the cytotoxicity of compounds **1**–**12** against four cancer cell lines, involving (7860), (HepG2), (H1975), and (Hela). The MTT assay was depicted as a previously used method [37]. The cells were seeded in complete medium per well within a 96-well plate. The cells were incubated at 37 °C for 12–24 h to facilitate adherent cell growth. Following this, compounds **1**–**12** solutions were added, and cells were cultured for 48 h. Subsequently, MTT solution was introduced into the wells and incubated for 4 h at 37 °C. DMSO was added to each well after the cell medium was discarded. The absorbance was measured at a wavelength of 570 nm.

### 3.7. Antimicrobial Assay

The antimicrobial assay against three bacteria (*Bacillus subtilis*, *Staphylococcus aureus*, and *Escherichia coli*) and one fungus (*Candida albicans*) was assessed for adopting the microbroth dilution reported previously [38]. The MIC was defined as the lowest concentration of the antimicrobial agent that completely inhibited the visual growth of an organism. Ciprofloxacin and amphotericin B were used as positive controls against bacteria and fungi, respectively.

### 3.8. Molecular Docking 

**Figure 4:**
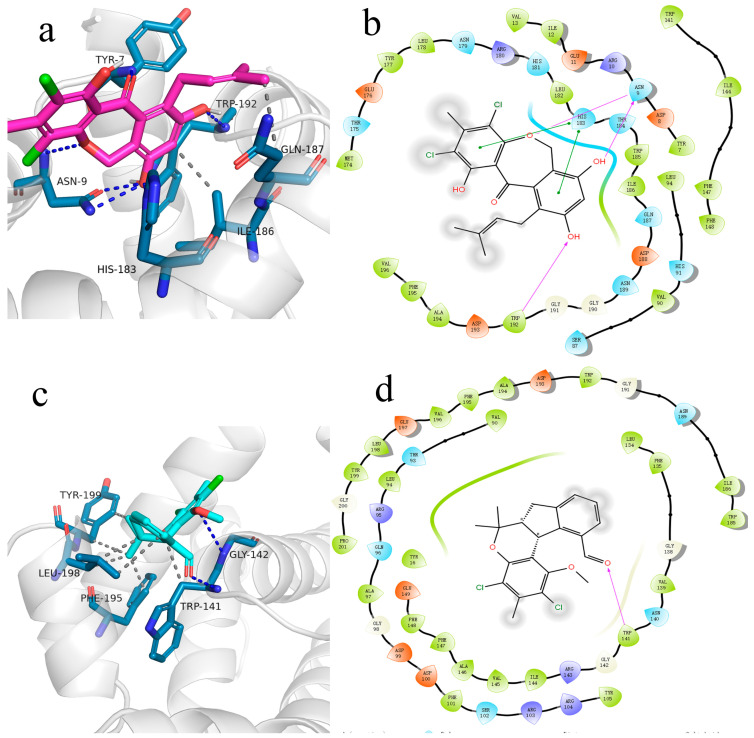
Representative docking poses of compounds **7** and **8** bound to Bcl-2 (PDB ID: 4LVT). The intermolecular interactions between Bcl-2 (PDB ID: 4LVT) and compounds **7** and **8** are as depicted in the maps (**a**,**c**) and the maps (**b**,**d**).

The molecular docking of compounds **7** and **8** was performed (Figure 4). The initial models for Bcl-2 (PDB ID:4LVT) was gained from the Protein Data Bank (http://www.rcsb.org, accessed on 16 November 2023). The 3D structures of compounds **7** and **8** were obtained from ChemBio 3D Ultra 14.0. AutoDock Vina (Center for Computational Structural Biology, La Jolla, the US, accessed on 16 November 2023), and AutoDockTools-1.5.6 were used to generate docking input files [39]. The docking results were then analyzed for interaction patterns using PyMOL 2.3.0.

## 4. Conclusions

In summary, we identified six new polyketide derivatives (**1**–**6**) and six known compounds (**7**–**12**) that were produced by chemical epigenetic cultivation. The absolute configurations of **1** and **2** were further established by circular dichroism (CD) cotton effects and the modified Mosher’s method. Compounds **7** and **8** manifested antibacterial activities against *Staphylococcus aureus* and *Bacillus subtilis*, with MIC values varying from 3 to 50 mg/mL. The result of the analysis showed that the potential to develop compound **7** as a lead drug with antibacterial activity is quite high. In addition, although the cytotoxic and antibacterial activities of compounds **7** and **8** were evaluated, the detailed mechanism of action is still undefined; thus, further studies are needed.

## Figures and Tables

**Table 1:** ^1^H and ^13^C NMR data of compounds **1**–**3** in CD_3_OD.

	1		2		3	
No.	*δ*_C_, Type	*δ*_H_ (*J* in Hz)	*δ*_C_, Type	*δ*_H_ (*J* in Hz)	*δ*_C_, Type	*δ*_H_ (*J* in Hz)
1	71.0, CH	5.04, dd (12.3, 2.7)	69.6, CH	5.08, dd (12.2, 2.6)	71.2, CH	5.06, dd (12.2, 2.7)
		4.95, d (12.3)		4.99, d (12.2)		4.96, d (12.2)
2	126.0, C		124.5, C		126.1, C	
3	151.1, C		149.9, C		151.2, C	
4	115.4, CH	6.60, d (8.1)	114.1, CH	6.63, d (8.0)	115.6, CH	6.61, d (8.2)
5	130.4, CH	6.87, d (8.1)	129.2, CH	6.88, d (8.0)	130.7, CH	6.89, d (8.2)
6	125.9, C		124.5, C		126.5, C	
7	143.4, C		141.6, C		142.8, C	
8	82.3, CH	5.53, brd (10.4)	83.4, CH	5.48, brd (10.2)	81.5, CH	5.51, m
9	39.1, CH_2_	1.83, ddd (14.3, 10.4, 2.0)	37.3, CH_2_	2.11, ddd (14.8, 3.9, 2.4)	39.7, CH_2_	1.87, ddd (14.5, 4.9, 2.4)
		1.62, ddd (14.3, 10.4, 2.2)		1.70, dd (14.8, 6.0)		1.90, ddd (14.5, 9.8, 7.1)
10	73.3, CH	3.71, ddd (10.4, 4.7, 2.1)	74.4, CH	3.69, m	76.5, CH	4.35, dd (9.9, 2.9)
11	71.7, CH	3.61, qd (6.4, 4.7)	69.9, CH	3.75, qd (6.2, 1.6)	213.5, C	
12	18.9, CH_3_	1.14, d (6.4)	17.3, CH_3_	1.19, d (6.2)	25.8, CH_3_	2.18, s
1’	31.0, CH_2_	3.27, dd (16.0, 7.0)	29.8, CH_2_	3.30, dd (16.0, 7.0)	31.2, CH_2_	3.24, dd (16.0, 6.9)
						3.35, dd (16.0, 7.4)
2’	128.8, CH	5.57, brt (7.2)	127.3, CH	5.54, d (7.1)	127.1, CH	5.30, t (7.6)
3’	132.3, C		131.0, C		136.6, C	
4’	14.3, CH_3_	1.75, s	12.6, CH_3_	1.75, s	21.5, CH_3_	1.80, s
5’	71.0, CH_2_	4.48, s	62.8, CH_2_	4.69, s	61.4, CH_2_	4.19, d (12.2),
						4.20, d (12.2),
7’	172.8, C		171.5, C			
8’	20.8, CH_3_	2.03, s	20.2, CH_3_	2.05, s		

**Table 2:** ^1^H and ^13^C NMR data of compounds **4**–**6** in CD_3_OD.

	4		5		6	
No.	*δ*_C_, Type	*δ*_H_ (*J* in Hz)	*δ*_C_, Type	*δ*_H_ (*J* in Hz)	*δ*_C_, Type	*δ*_H_ (*J* in Hz)
1	71.4, CH	5.05, dd (14.3, 2.2)	70.1, CH	5.06, dd (12.3, 2.5)	69.6, CH	5.03, dd (12.2, 2.3)
		4.95, d (14.3)		4.96, d (12.3)		4.92, d (12.2)
2	126.1, C		125.0, C		124.7, C	
3	151.1, C		149.7, C		149.7, C	
4	115.7, CH	6.63, d (8.2)	114.3, CH	6.62, d (8.1)	114.1, CH	6.60, d (8.1)
5	130.8, CH	6.93, d (8.2)	129.3, CH	6.89, d (8.1)	129.2, CH	6.87, d (8.1)
6	126.4, C		125.6, C	5.31, t (7.8)	125.5, CH	5.31, t (7.8)
7	142.0, C		141.4, C		140.6, C	
8	81.2, CH	5.76, m	79.9, CH	5.74, m	79.4, CH_2_	5.72, m
9	44.3, CH_2_	2.91, d (9.6)	43.1, CH_2_	2.88, m	38.6, CH_2_	2.23, m
		2.85, dd (16.2, 2.0)				
10	74.4, CH	4.23, q (7.0)	211.8, C		211.5, CH	
11	212.7, C		74.7, CH	4.28, q (7.0)	73.3, CH	4.24, q (7.0)
12	19.5, CH_3_	1.29, d (7.0)	18.1, CH_3_	1.29, d (7.0)	17.9, CH_3_	1.33, d (7.0)
1’	31.2, CH_2_	3.21, m, 3.25, m	29.6, CH_2_	3.30, d (7.1)	29.6, CH_2_	3.30, d (7.1)
2’	124.8, CH	5.46, t (7.0)	125.0, CH	5.20, t (7.1)	125.3, CH	5.22, t (7.1)
3’	137, C		135.4, C		135.2, C	
4’	13.9, CH_3_	1.71, s	20.2, CH_3_	1.81, s	20.1, CH_3_	1.83, s
5’	68.6, CH_2_	3.95, s	60.0, CH_2_	4.16, s	60.0, CH_2_	4.16, s

**Table 3:** Antibacterial activities and cytotoxicity of **1**–**12**.

Comp.	Cytotoxicity (IC_50_ in μM)				Antibacterial Activities (MIC μg/mL)			
	H1975	7860	Hela	HepG2	*B. subtilis*	*S. aureas*	*E. coli*	*C. albicans*
**1**	>100	>100	>100	>100	>100	>100	>100	>100
**2**	>100	>100	>100	>100	>100	>100	>100	>100
**3**	>100	>100	>100	>100	>100	>100	>100	>100
**4**	>100	>100	>100	>100	>100	>100	>100	>100
**5**	>100	>100	>100	>100	>100	>100	>100	>100
**6**	>100	>100	>100	>100	>100	>100	>100	>100
**7**	83.6	>100	63.5	16.2	3.0	3.0	>100	>100
**8**	>100	63.1	45.0	34.8	50.0	50.0	50.0	>100
**9**	>100	>100	>100	>100	>100	>100	>100	>100
**10**	>100	>100	>100	>100	>100	>100	>100	>100
**11**	>100	>100	>100	>100	>100	>100	>100	>100
**12**	>100	>100	>100	>100	>100	>100	>100	>100
Adriamycin	1.48	2	2.6	2.2				
^a^ Ciprofloxacin					0.25 ^a^	0.13 ^a^	0.13 ^a^	
^b^ Amphotericin								1.0 ^b^

^a^, Ciprofloxacin was used as a positive control against bacteria; ^b^, Amphotericin was used as a positive control against fungi.

## Data Availability

The data presented in this study are available in the main text and the Supplementary Materials of this article.

## Supplementary Materials

The following supporting information can be downloaded at https://www.mdpi.com/article/10.3390/md22010015/s1, Figures S1–S35: 1D, 2D NMR, and HRESIMS spectra of compounds **1**–**6**. Figures S36,S37: ^1^H NMR spectrum of the compounds 2a,2b; Figure S38: The Δδ (*δ*S–*δ*R) values from the (*S*)- and (*R*)-MTPA esters of 2.
