# Supplementary material for: Pestalotiopols E–J, Six New Polyketide Derivatives from a Marine Derived Fungus Pestalotiopsis sp. SWMU-WZ04-1
Source: Mar Drugs. 2023 Dec 26;22(1):15. doi: 10.3390/md22010015 (PMC10820063; doi:10.3390/md22010015)
Supplement: Supplementary file 1 [file marinedrugs-22-00015-s001.zip › marinedrugs-2772179-supplementary.pdf]

Supporting Information

Pestalotiopols E-J, Six New Polyketide Derivatives from a Marine Derived Fungus *Pestalotiopsis* sp. SWMU-WZ04-1

Liyuan Jiang [1], Baorui Teng [1], Mengyu, Zhang [1], Siwei Chen [1], Dan Zhang [1],

Longfei Zhai [2], Jiafu Lin [3],*, Hui Lei [1]*

[1] *School of Pharmacy, Southwest Medical University, Luzhou 646000, China,*

[2] *Antibiotics Research and Re-evaluation Key Laboratory of Sichuan Province, Sichuan Industrial Institute of Antibiotics, Chengdu University, Chengdu 610106, China,*

[3] *School of Pharmacy, Chengdu University, Chengdu 610106, China,*

Corresponding author.

E-mail addresses: linjiafu@cdu.edu.cn (J.L.); huilei@swmu.edu.cn (H.L.)

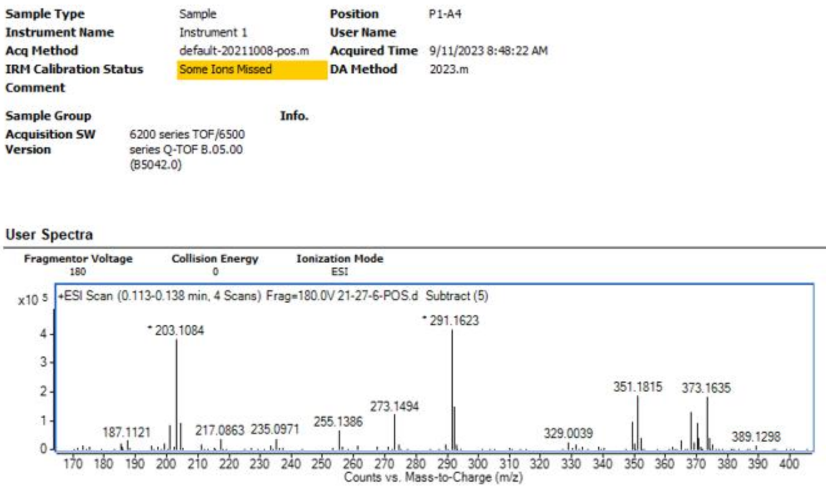

Figure S1 HRESI-MS spectrum of the compound **1**

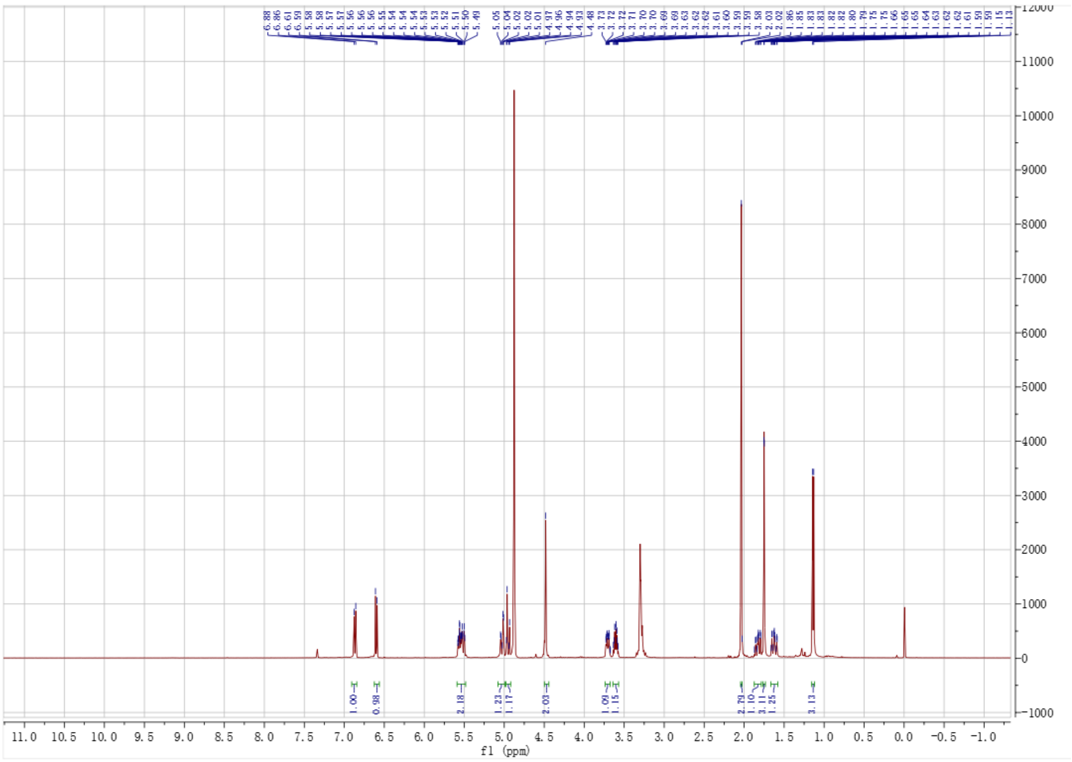

Figure S2 ^{1}H NMR (600 MHz, CD_3OD) spectrum of the compound **1**

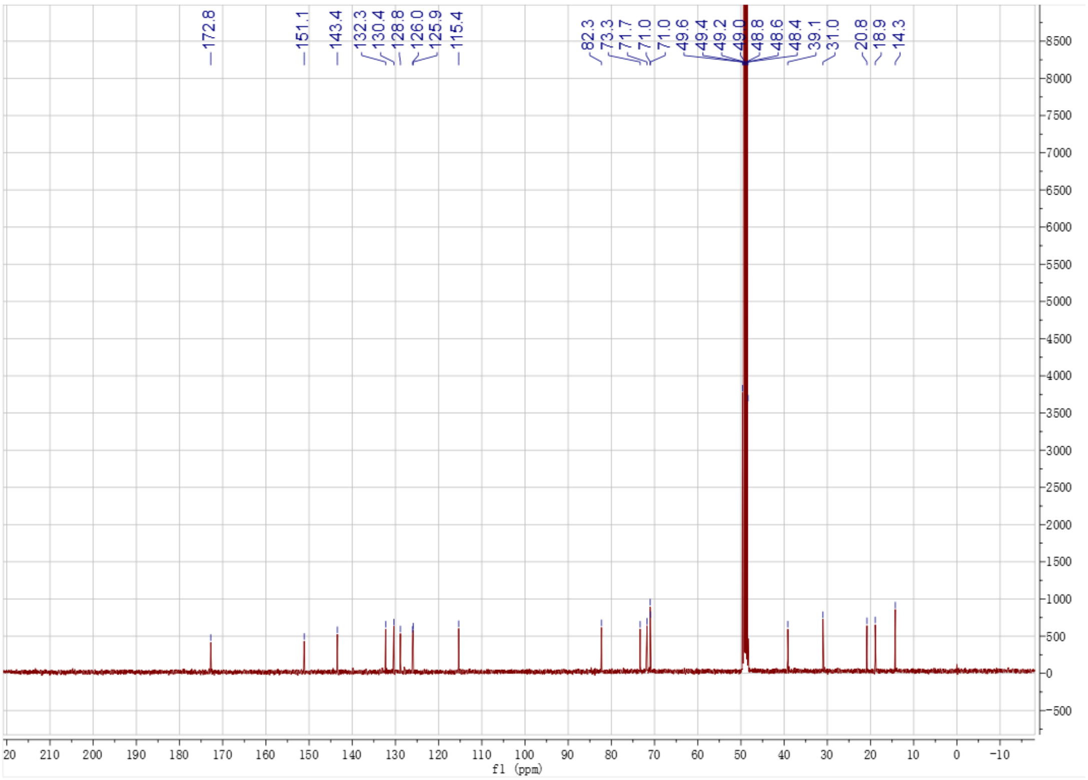

Figure S3 ^{13}C NMR (150 MHz, CD_3OD) spectrum of the compound **1**

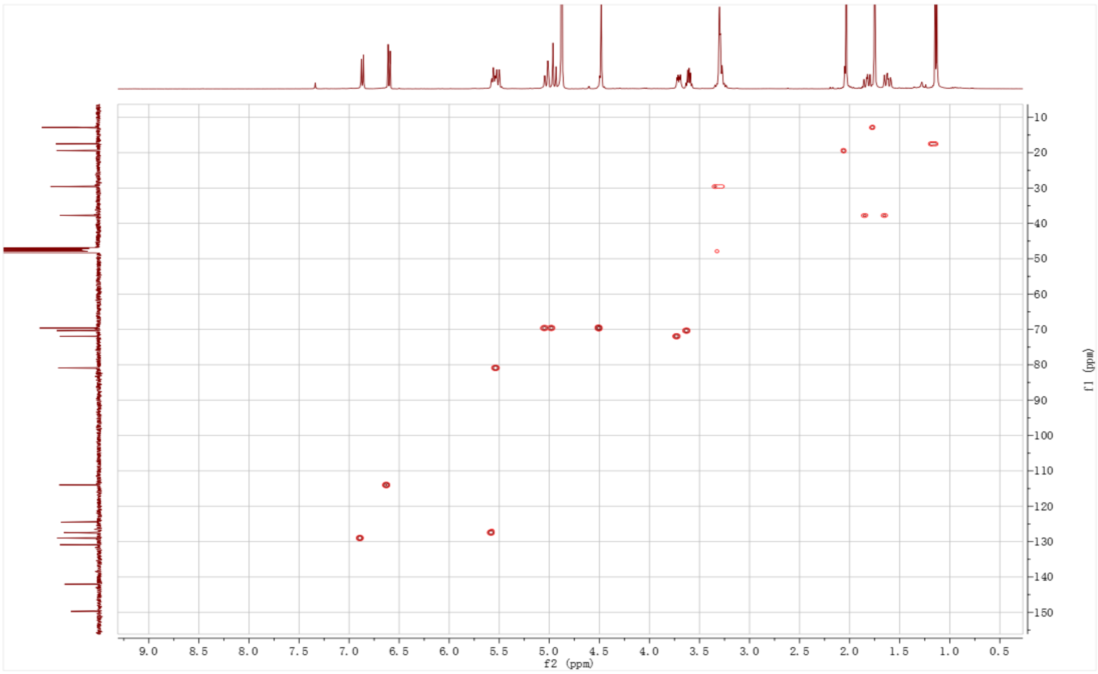

Figure S4 HSQC spectrum of the compound **1** (CD_3OD)

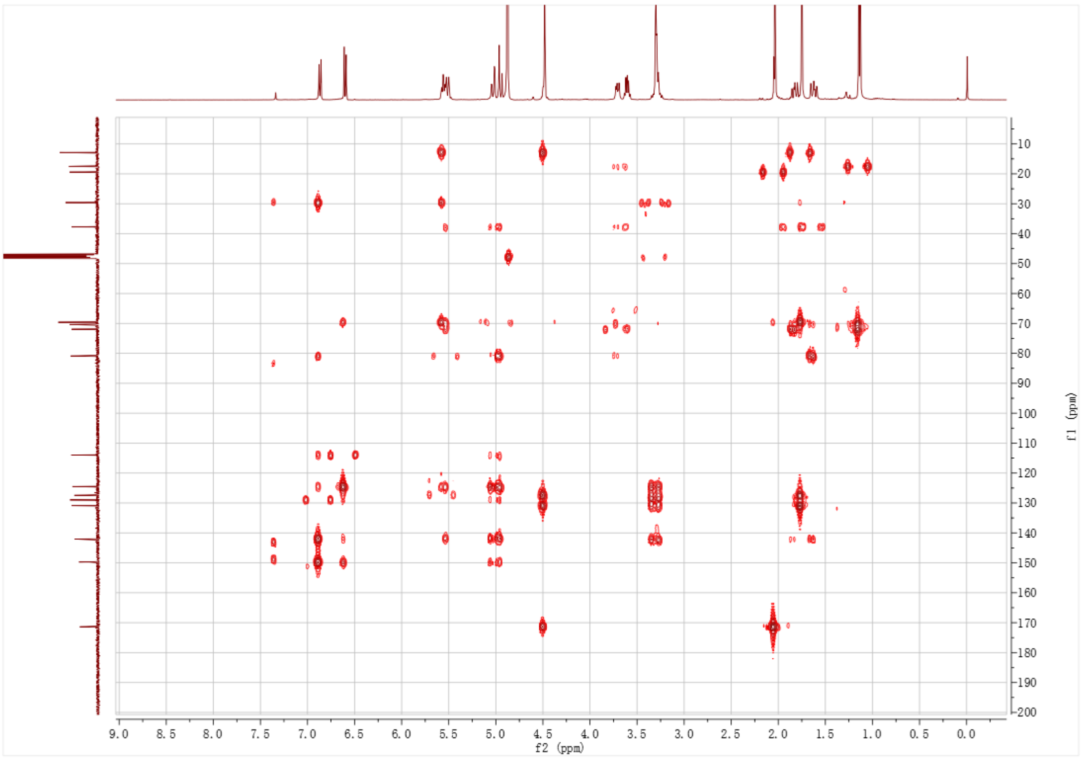

Figure S5 HMBC spectrum of the compound **1**(CD_3OD)

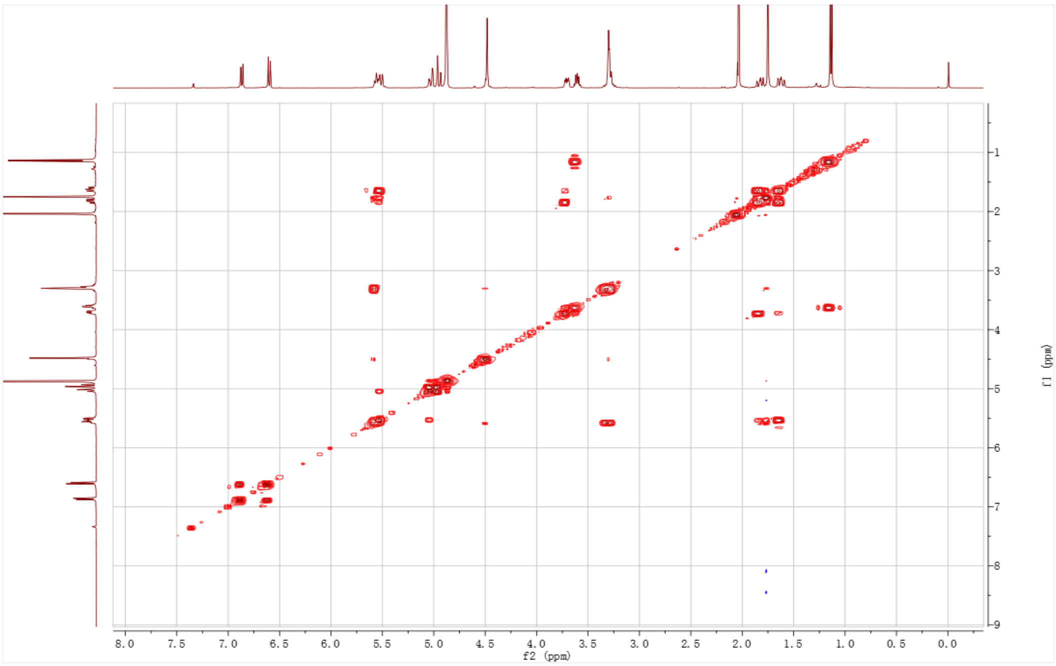

Figure S6 COSY spectrum of the compound **1**(CD_3OD)

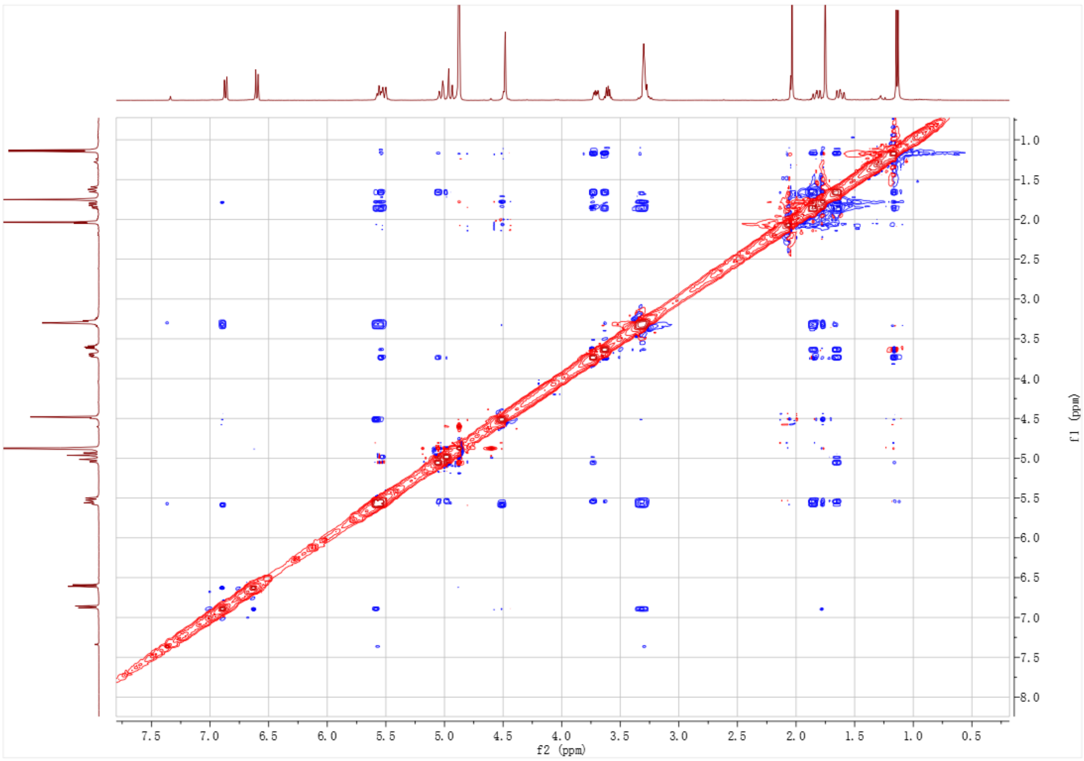

Figure S7 NOESY spectrum of the compound **1**(CD_3OD)

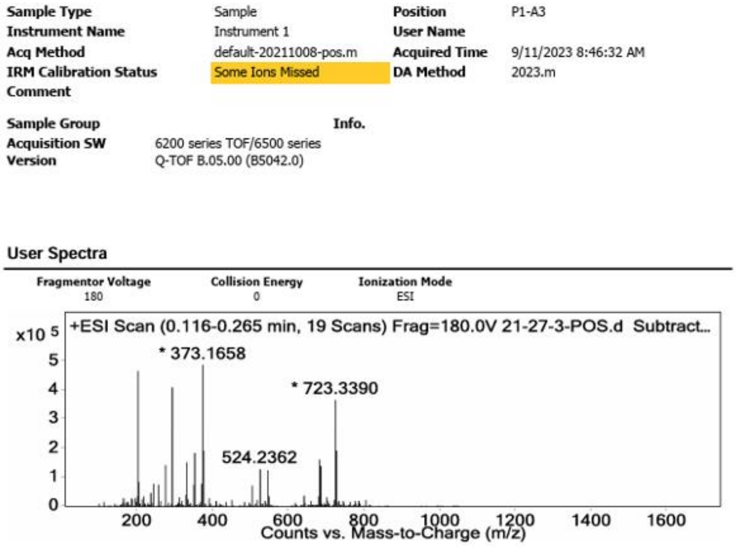

Figure S8 HRESI-MS spectrum of the compound **2**

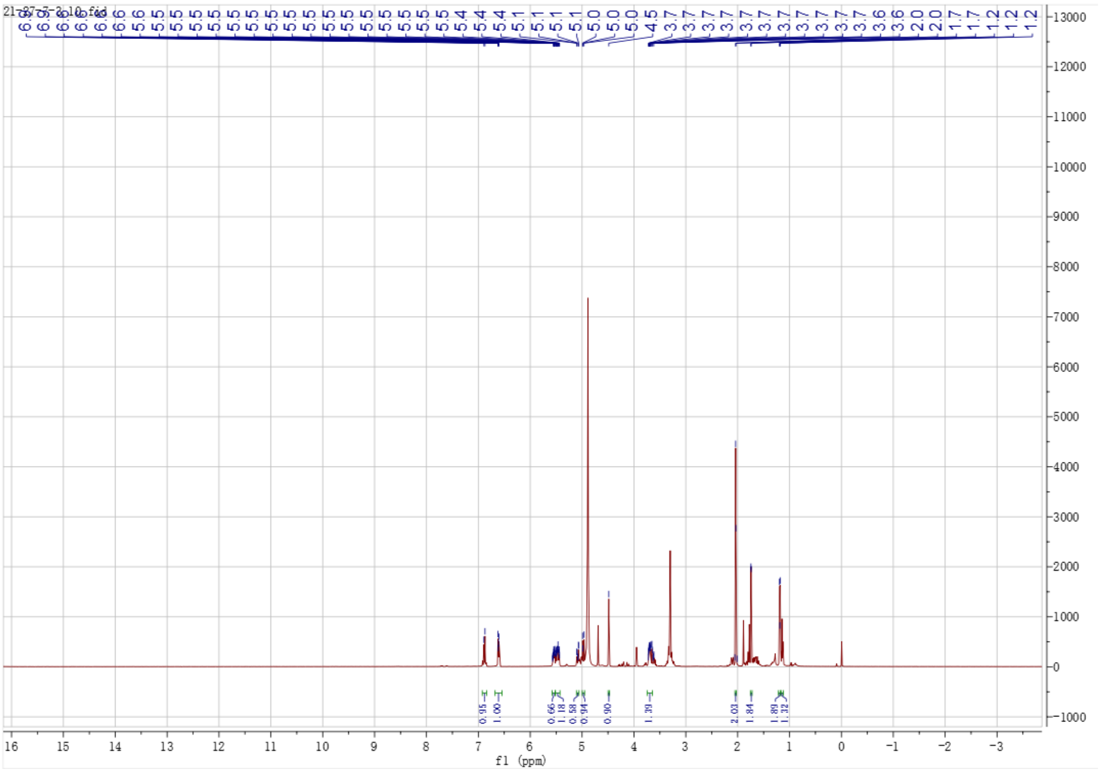

Figure S9 ^{1}H NMR (600 MHz, CD_3OD) spectrum of the compound **2**

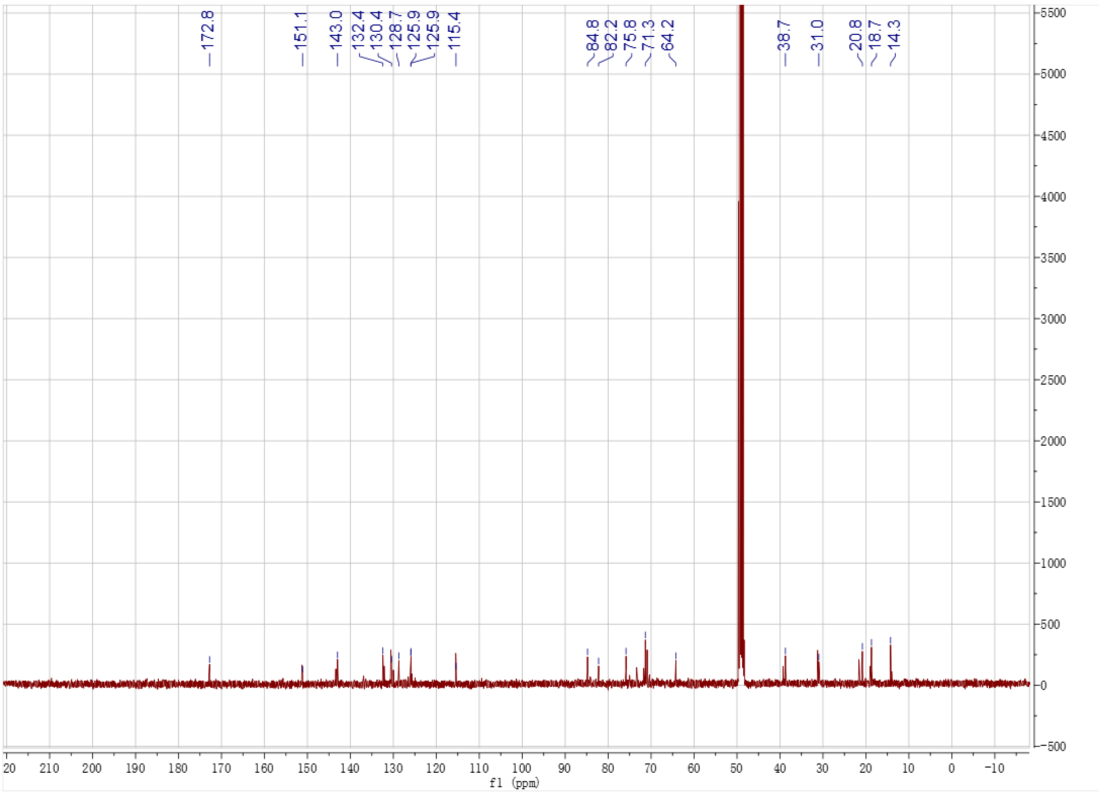

Figure S10 ^{13}C NMR (150 MHz, CD_3OD) spectrum of the compound **2**

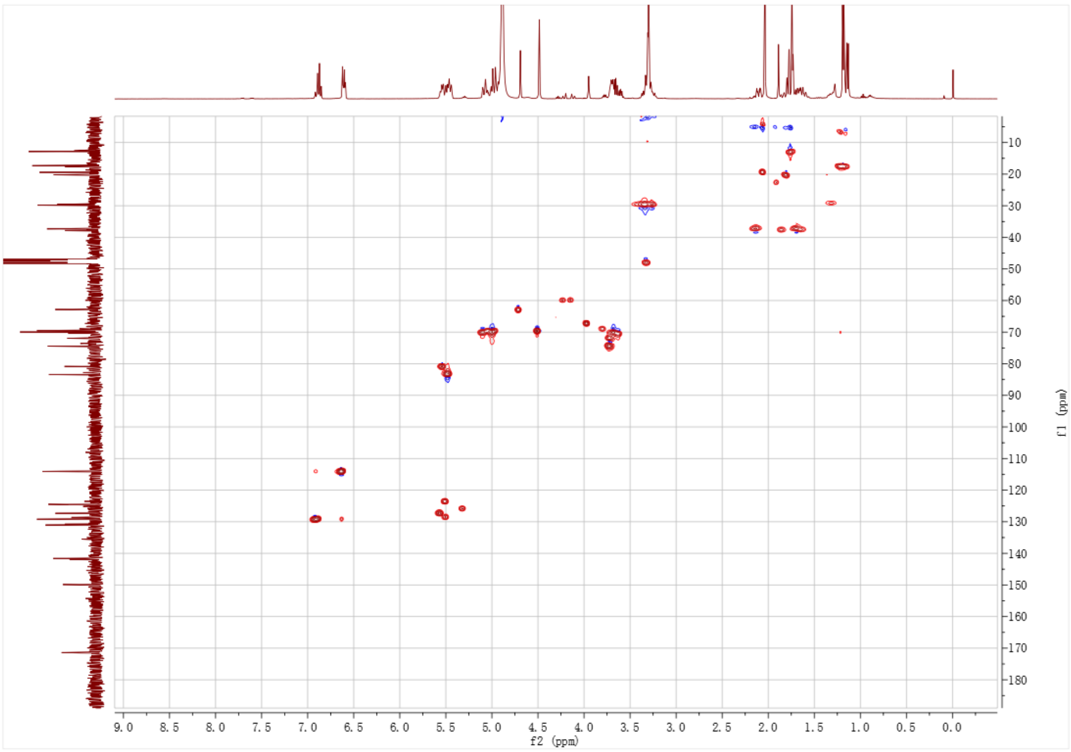

Figure S11 HSQC spectrum of the compound **2** (CD_3OD)

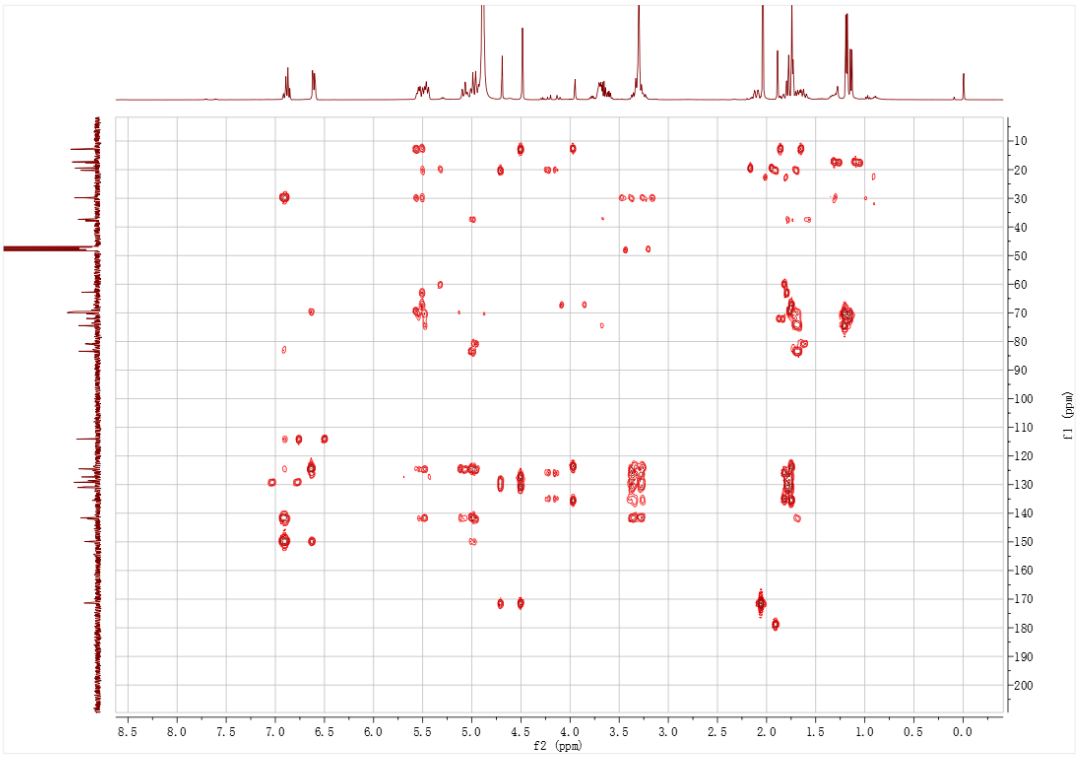

Figure S12 HMBC spectrum of the compound **2** (CD_3OD)

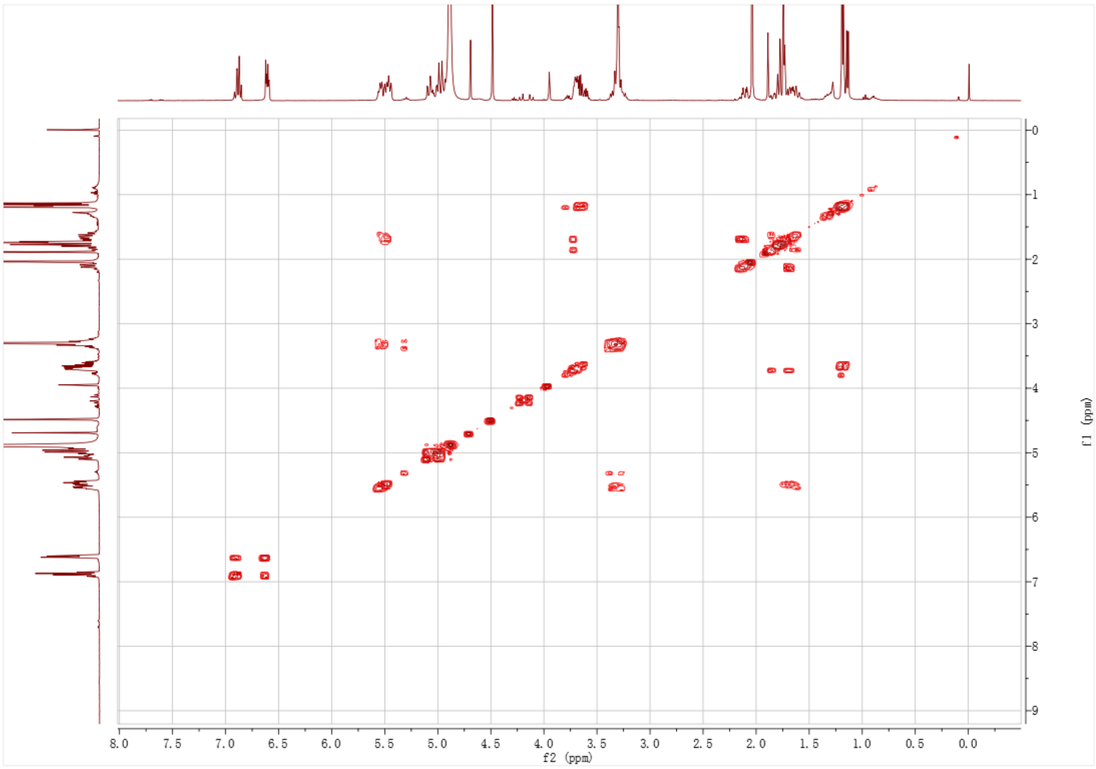

Figure S13 COSY spectrum of the compound **2** (CD_3OD)

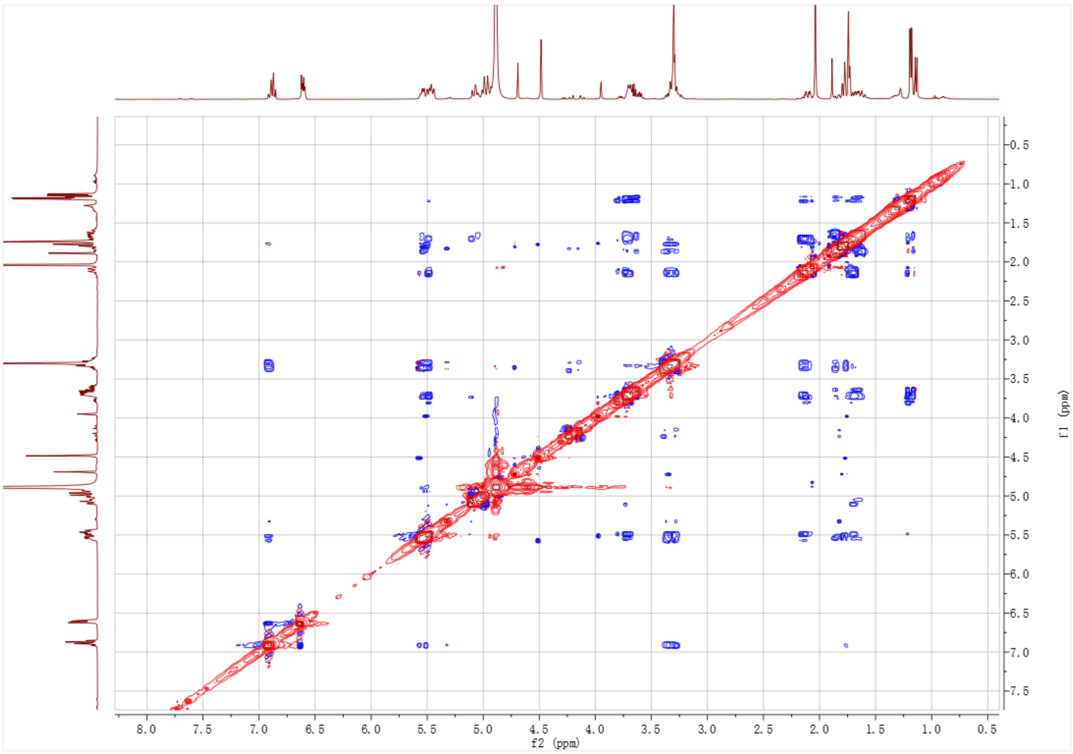

Figure S14 NOESY spectrum of the compound **2** (CD_3OD)

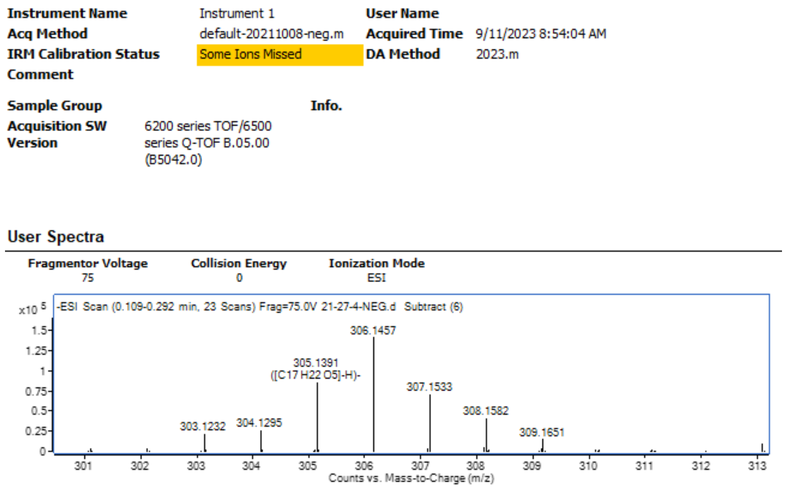

Figure S15 HRESI-MS spectrum of the compound **3**

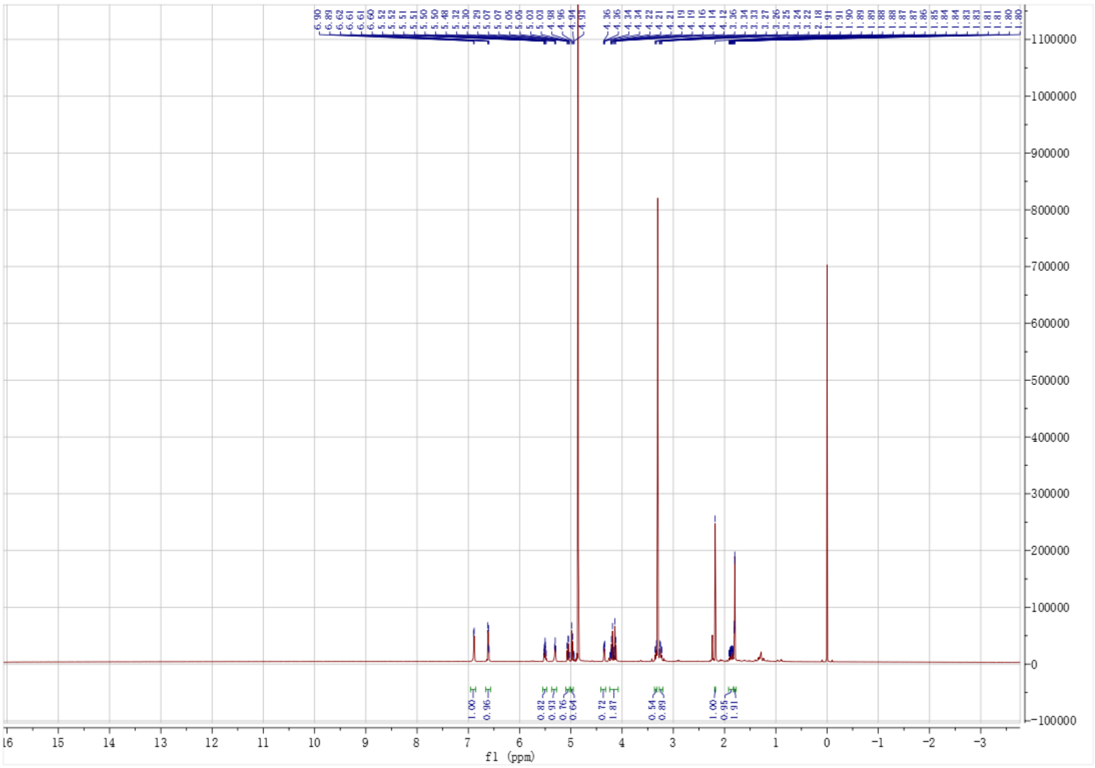

Figure S16 ^{1}H NMR (600 MHz, CD_3OD) spectrum of the compound **3**

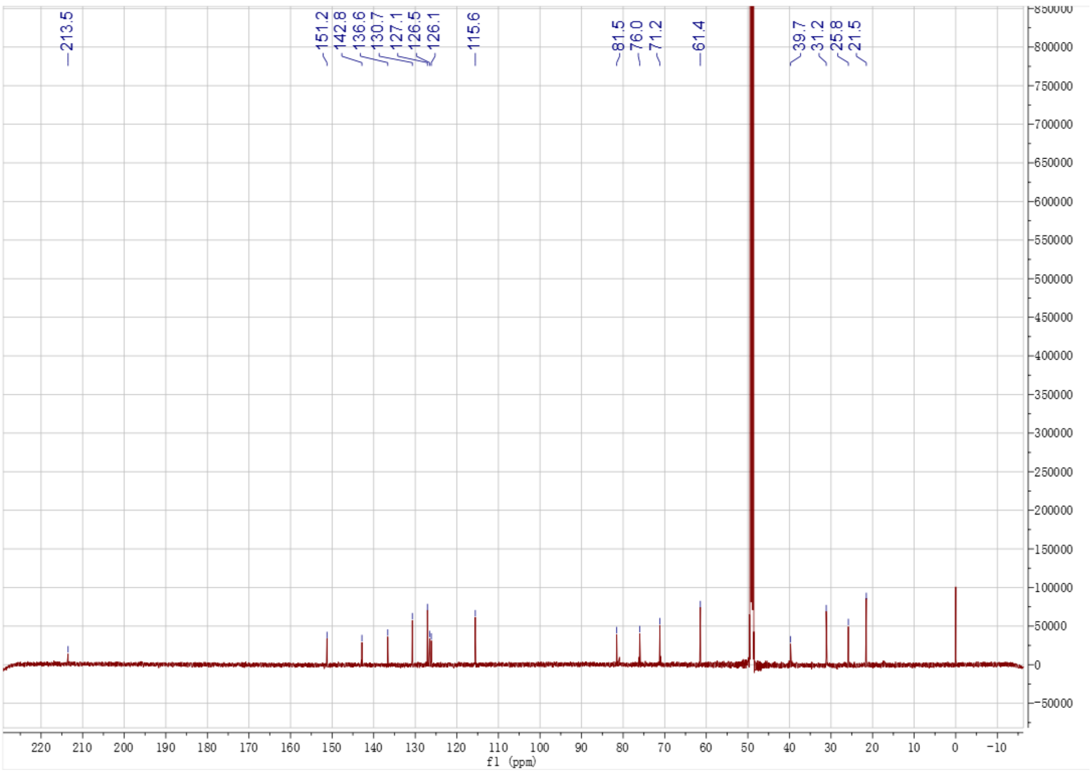

Figure S17 ^{13}C NMR (150 MHz, CD_3OD) spectrum of the compound **3**

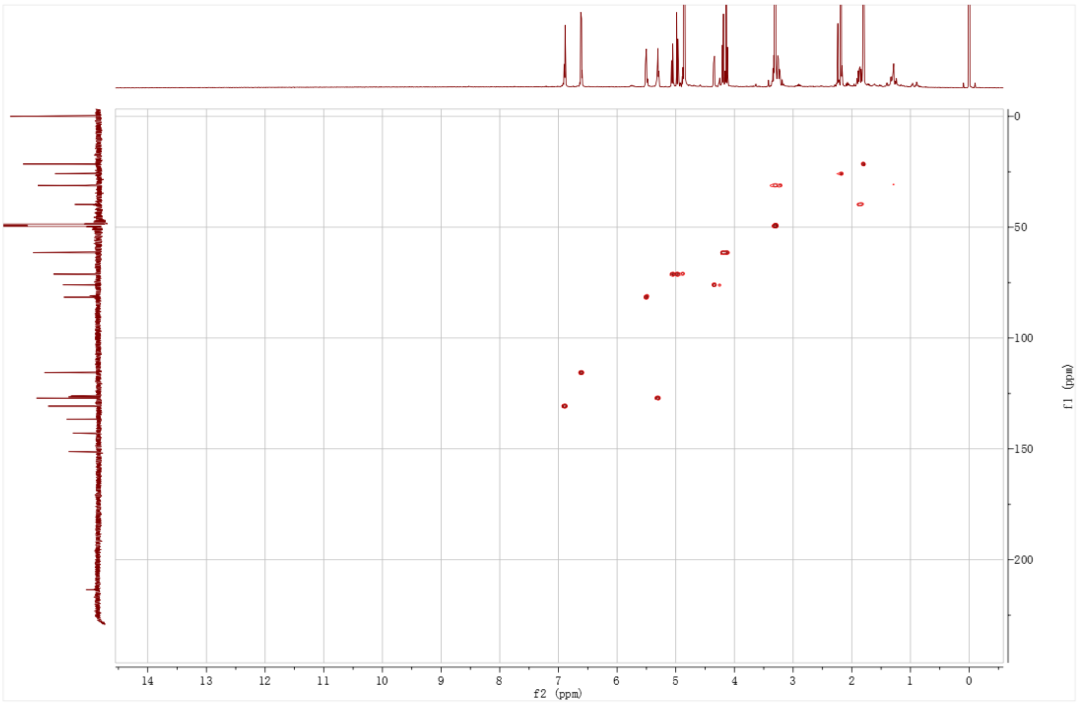

Figure S18 HSQC spectrum of the compound **3** (CD_3OD)

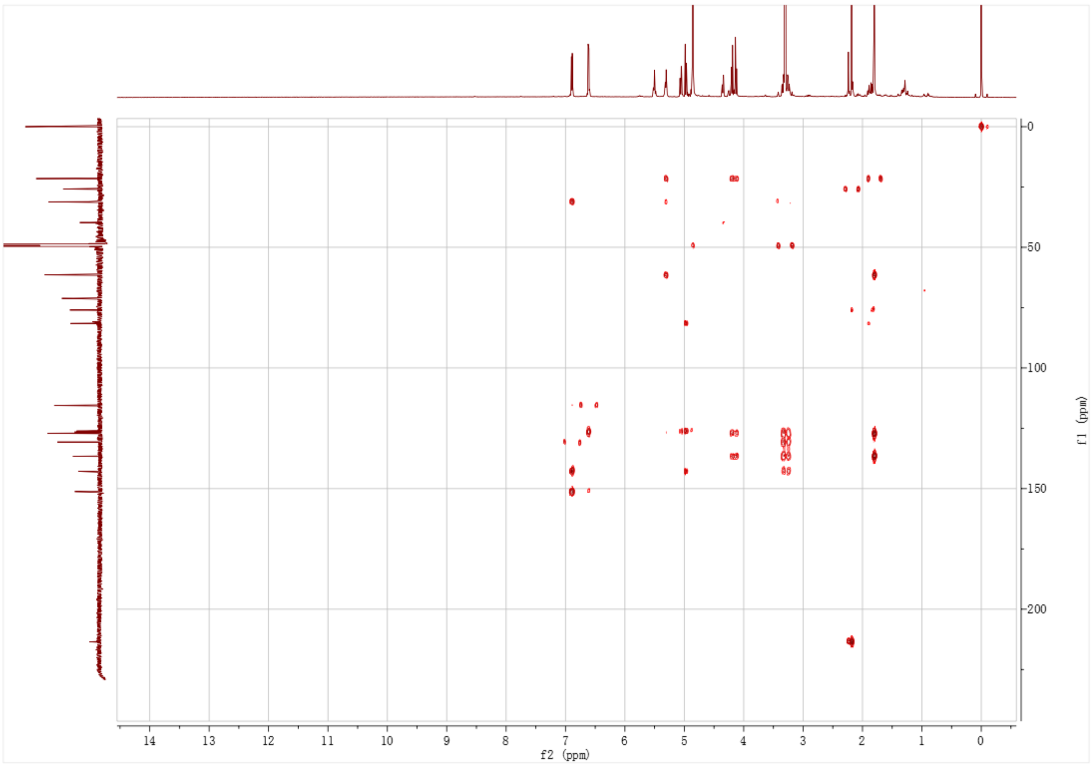

Figure S19 HMBC spectrum of the compound **3** (CD_3OD)

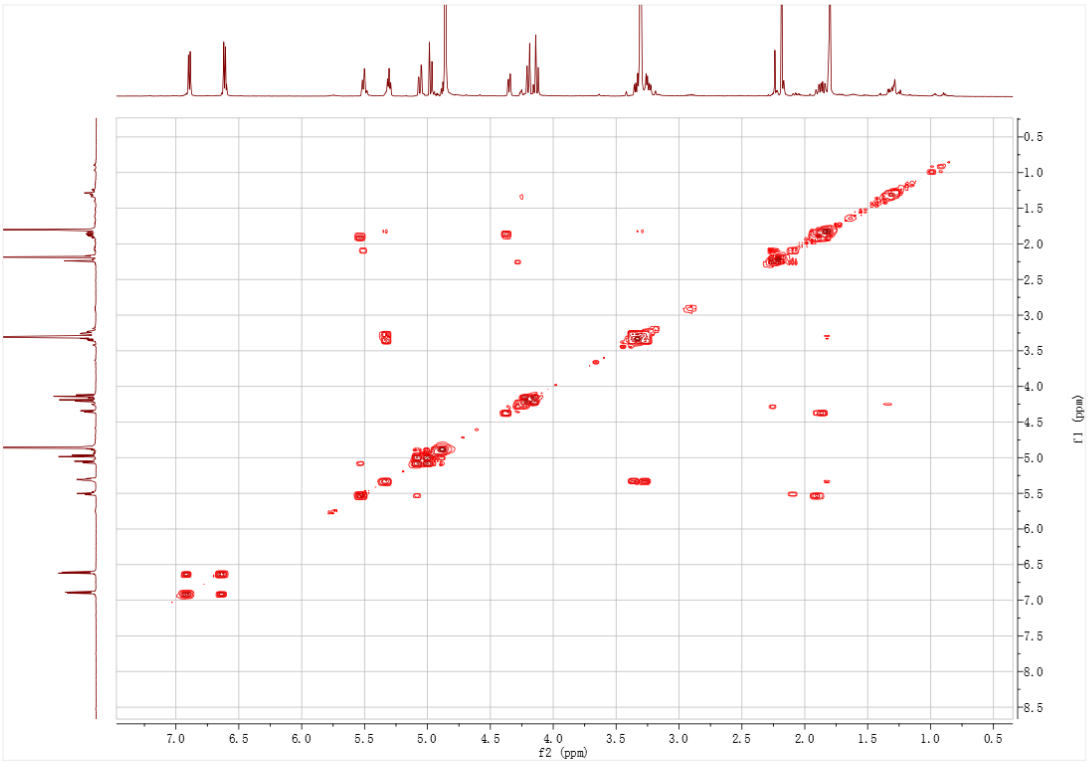

Figure S20 COSY spectrum of the compound **3** (CD_3OD)

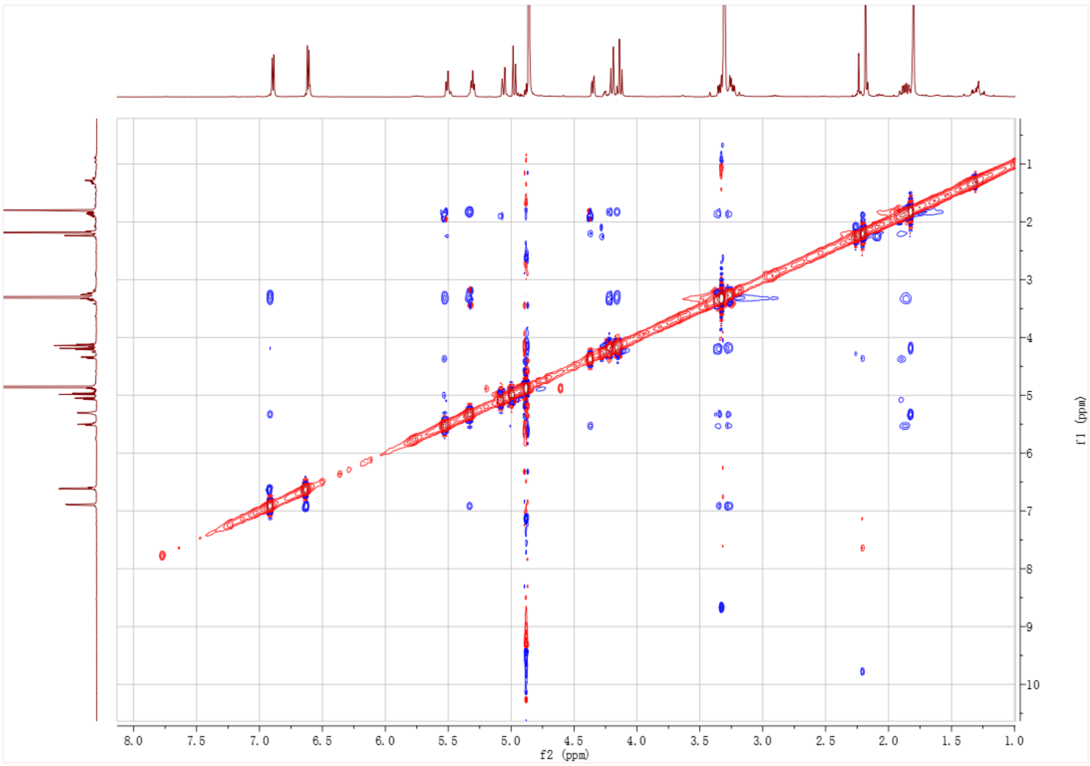

Figure S21 NOESY spectrum of the compound **3** (CD_3OD)

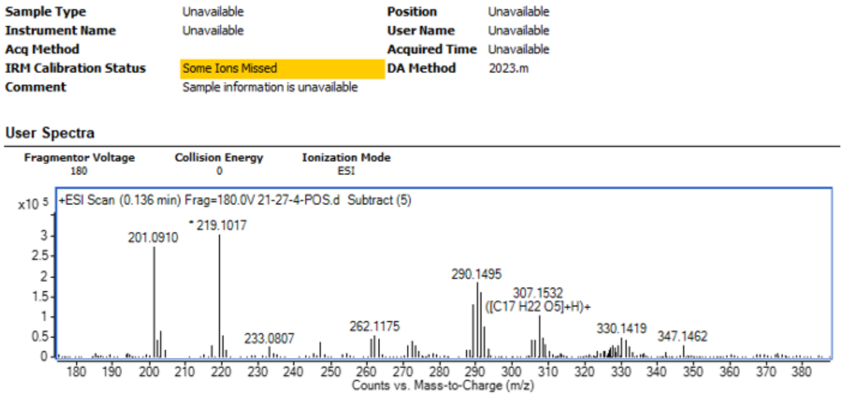

Figure S22 HRESI-MS spectrum of the compound **4**

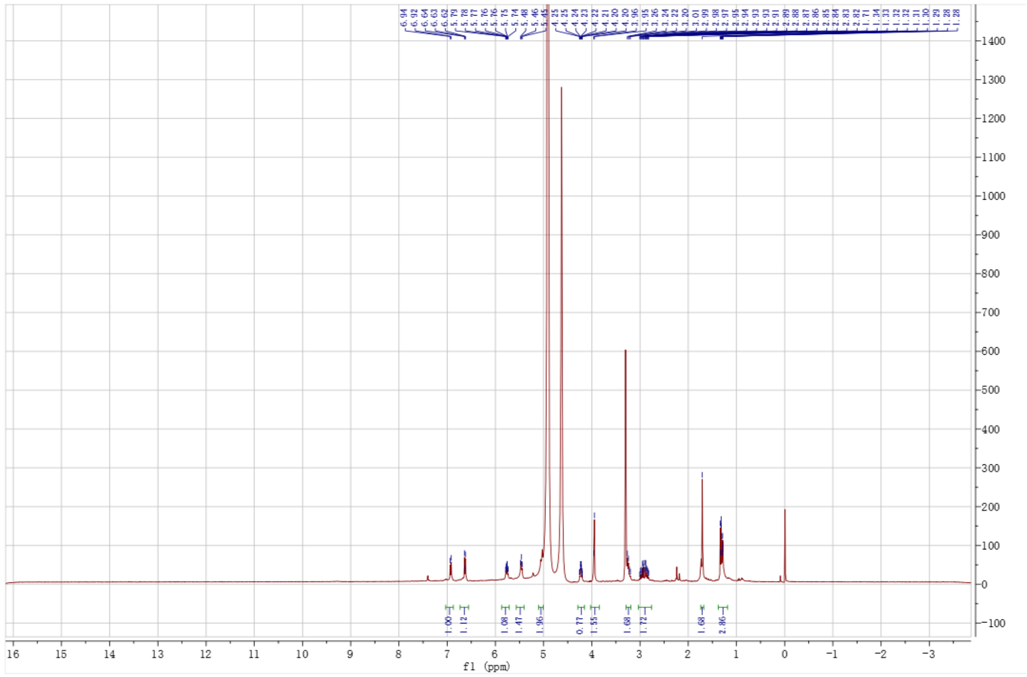

Figure S23 ^{1}H NMR (600 MHz, CD_3OD) spectrum of the compound **4**

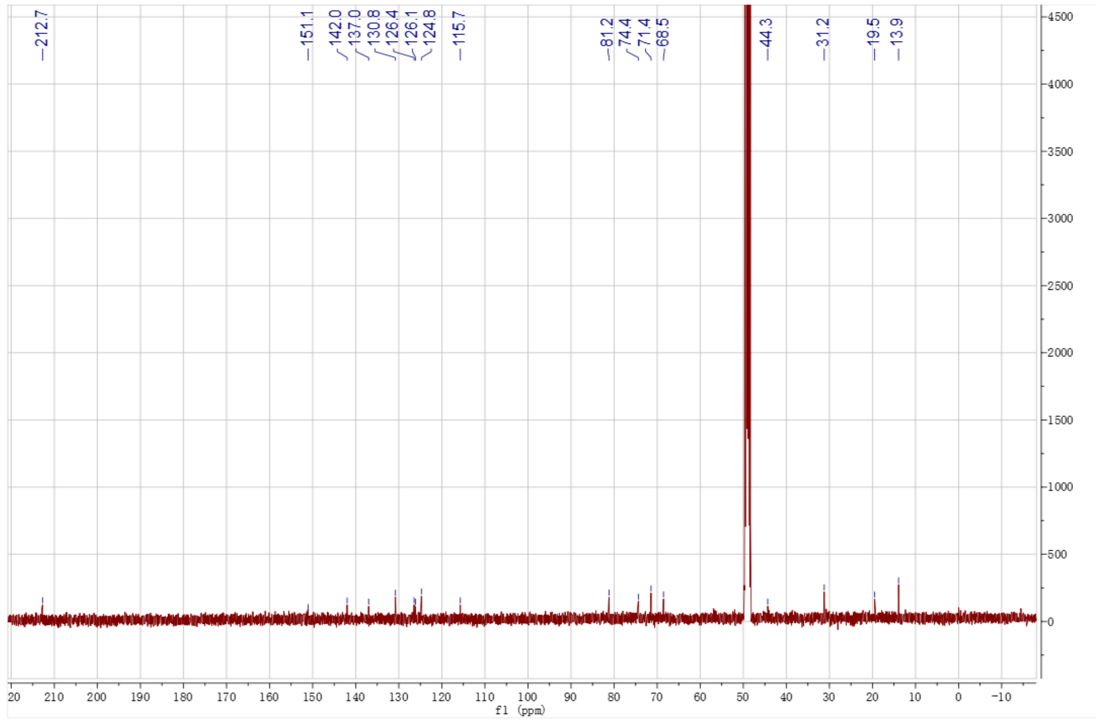

Figure S24 ^{13}C NMR (150 MHz, CD_3OD) spectrum of the compound **4**

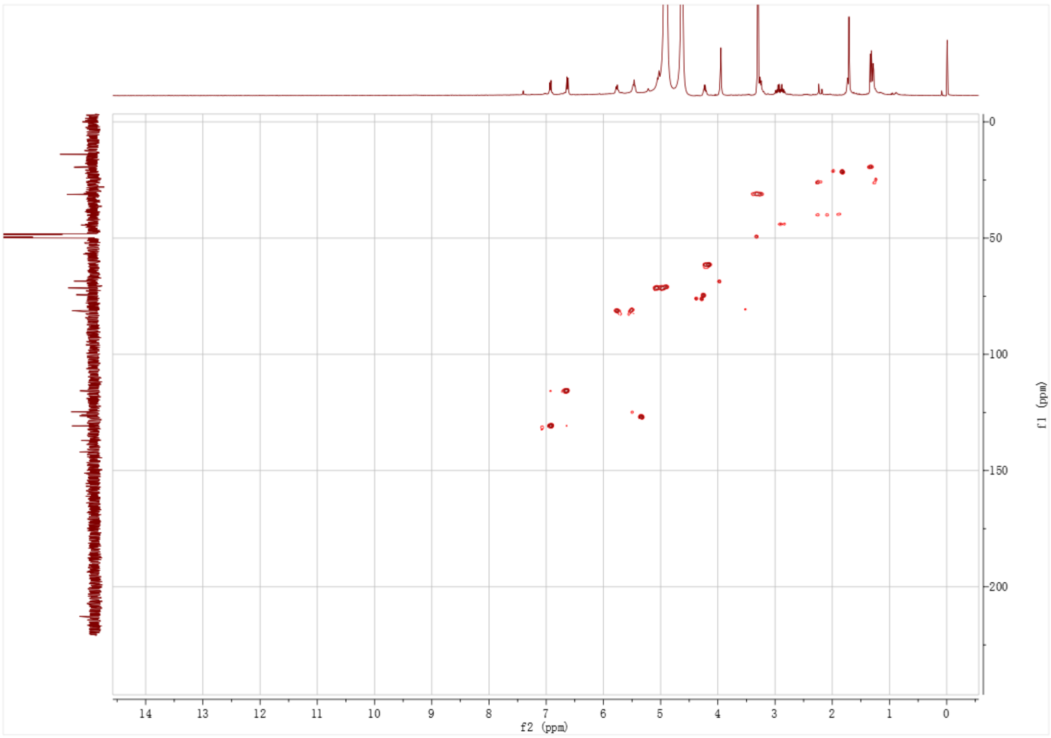

Figure S25 HSQC spectrum of the compound **4** (CD_3OD)

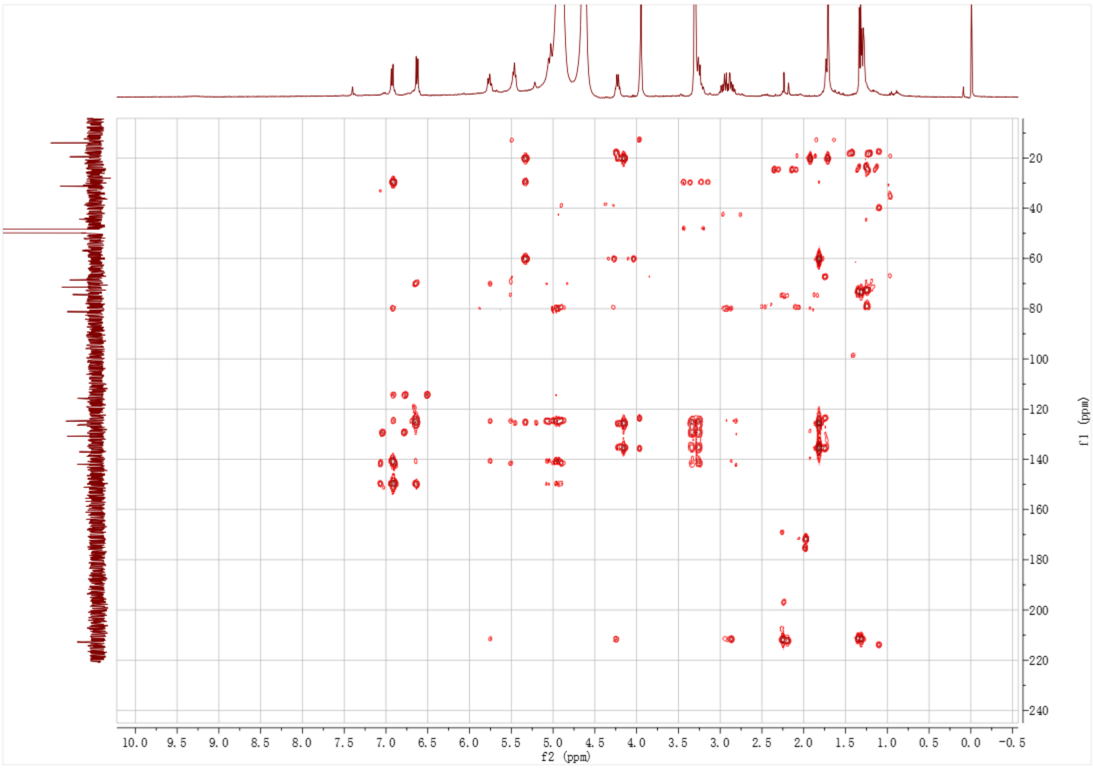

Figure S26 HMBC spectrum of the compound **4** (CD_3OD)

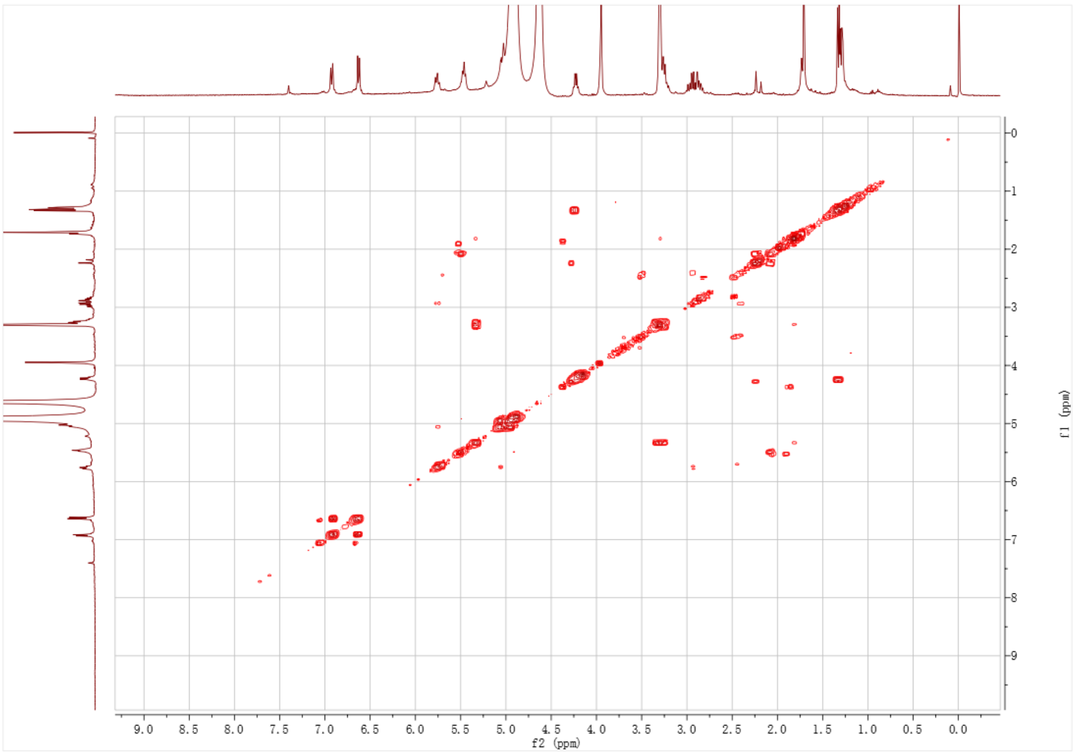

Figure S27 COSY spectrum of the compound **4** (CD_3OD)

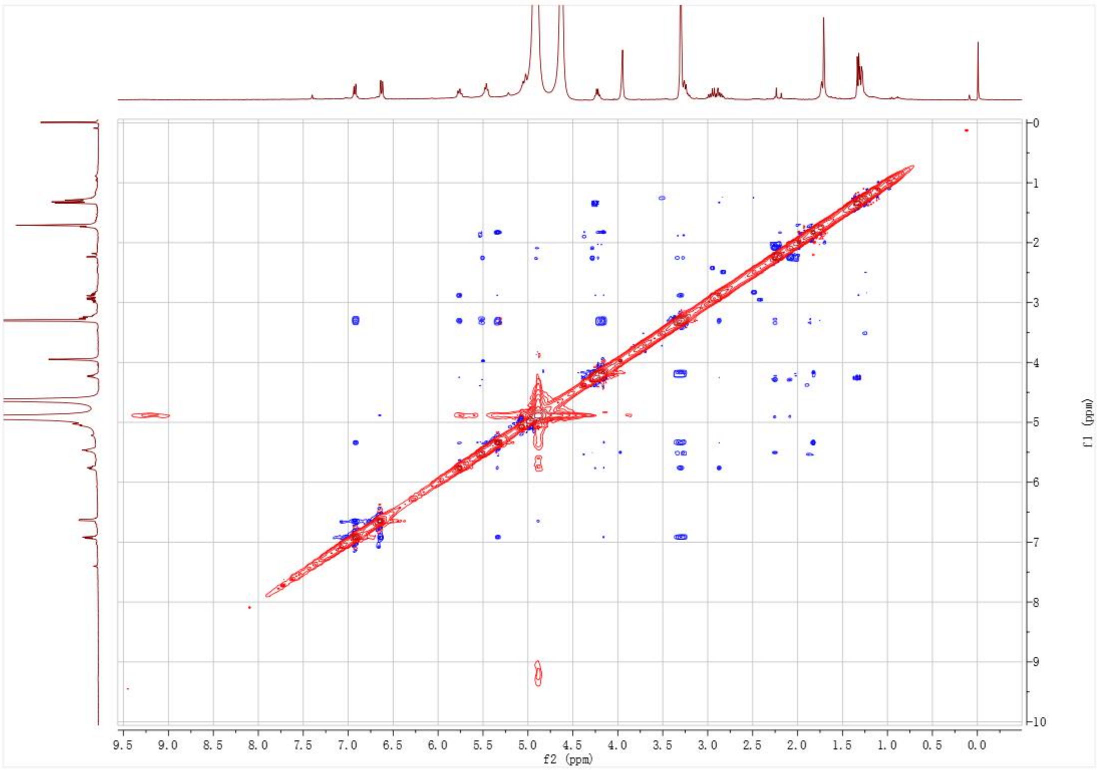

Figure S28 NOESY spectrum of the compound **4** (CD_3OD)

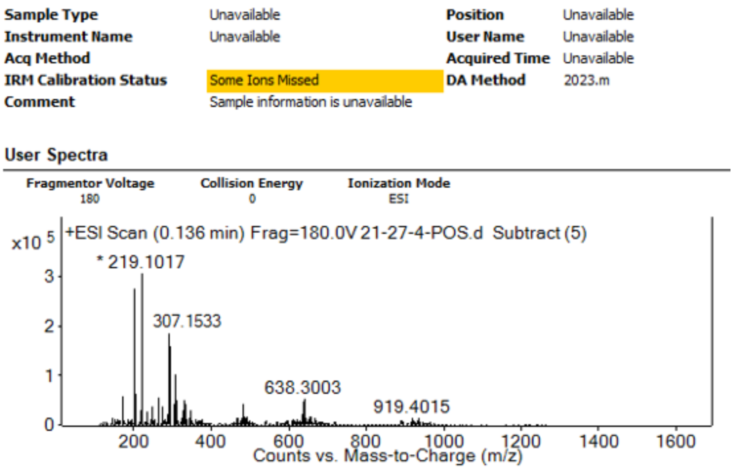

Figure S29 HRESI-MS spectrum of the compounds **5/6**

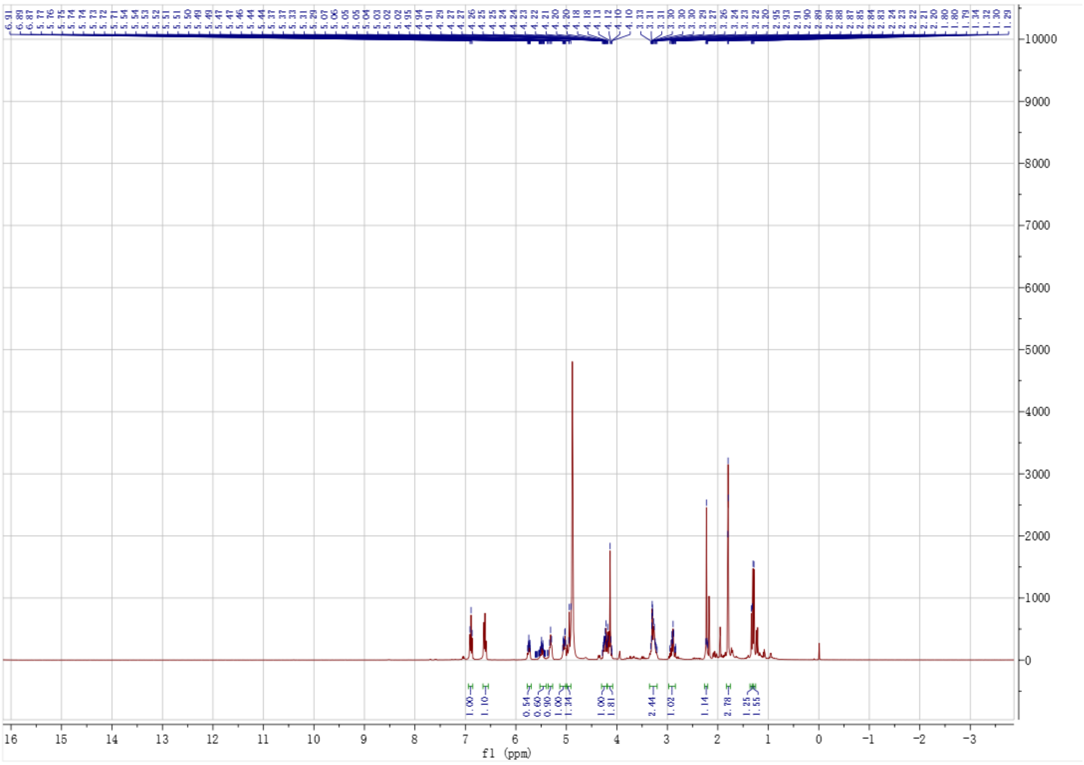

Figure S30 ^{1}H NMR (600 MHz, CD_3OD) spectrum of the compounds **5/6**

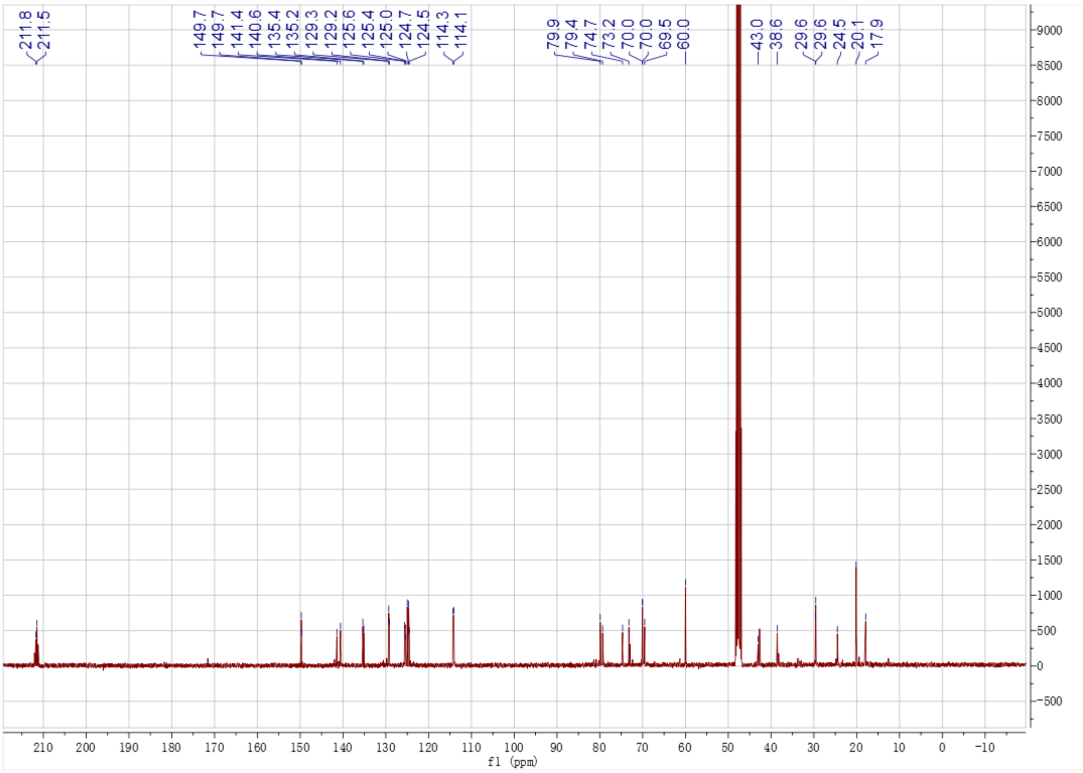

Figure S31 ^{13}C NMR (150 MHz, CD_3OD) spectrum of the compounds **5/6**

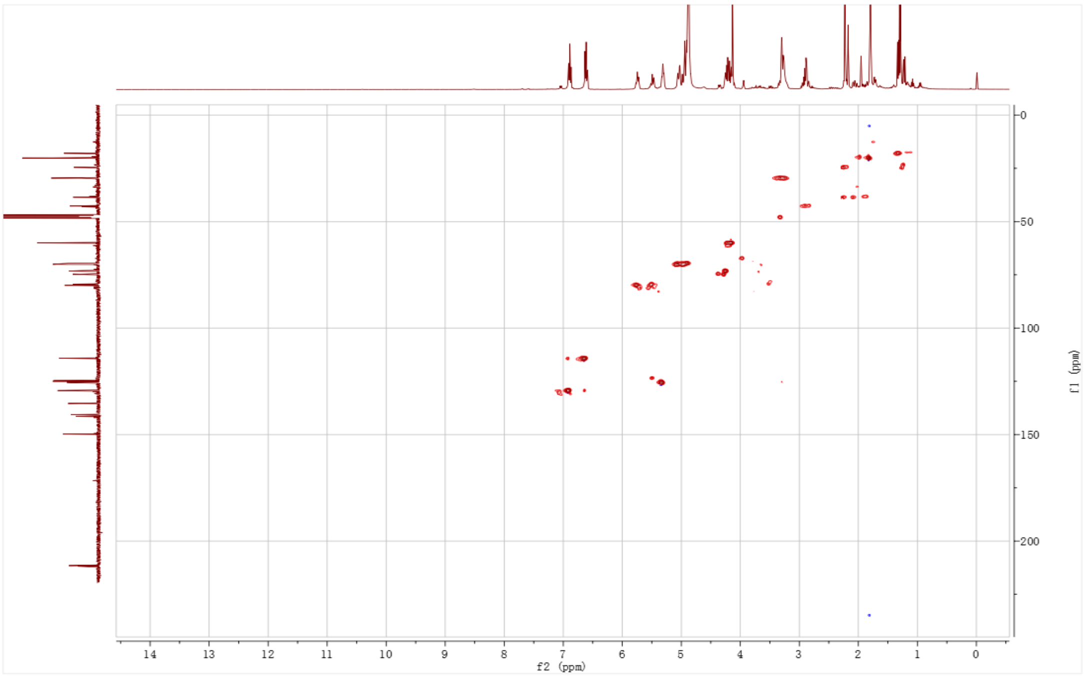

Figure S32 HSQC spectrum of the compounds **5/6** (CD_3OD)

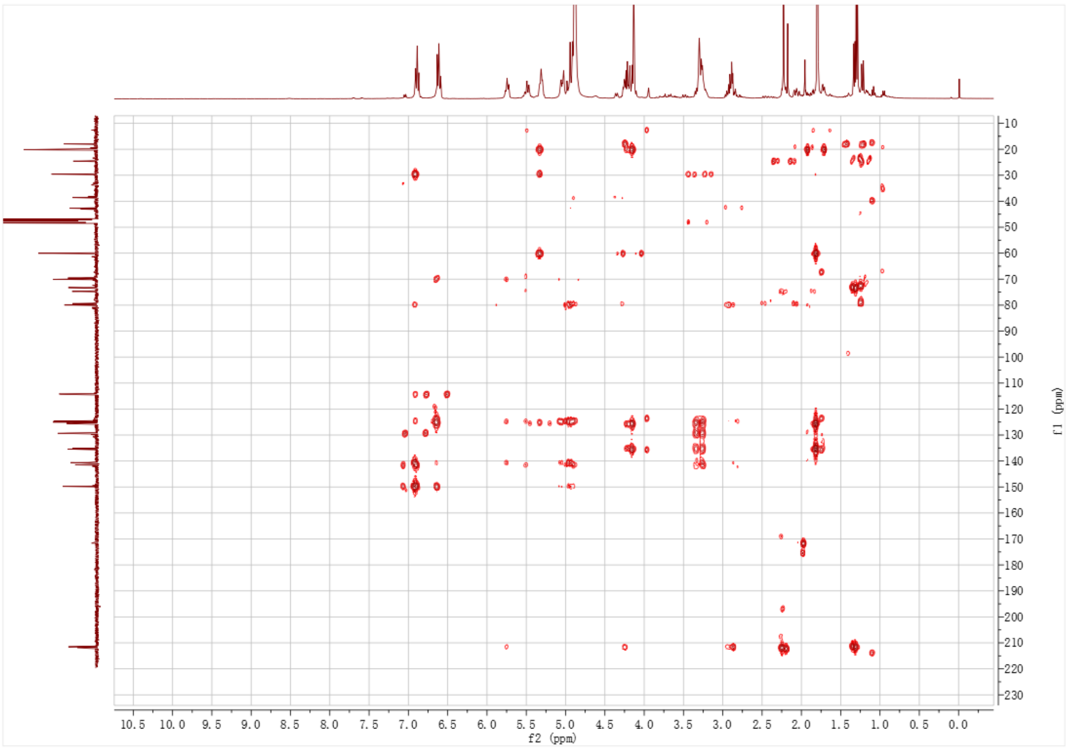

Figure S33 HMBC spectrum of the compounds **5/6** (CD_3OD)

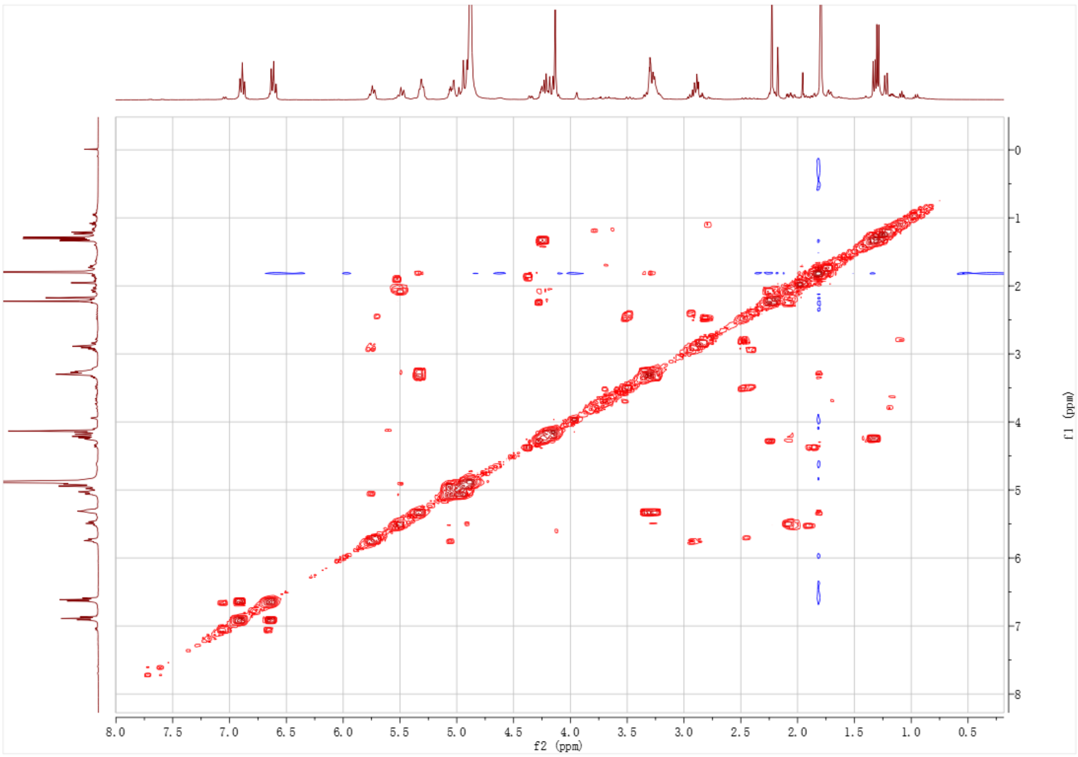

Figure S34 COSY spectrum of the compounds **5/6** (CD_3OD)

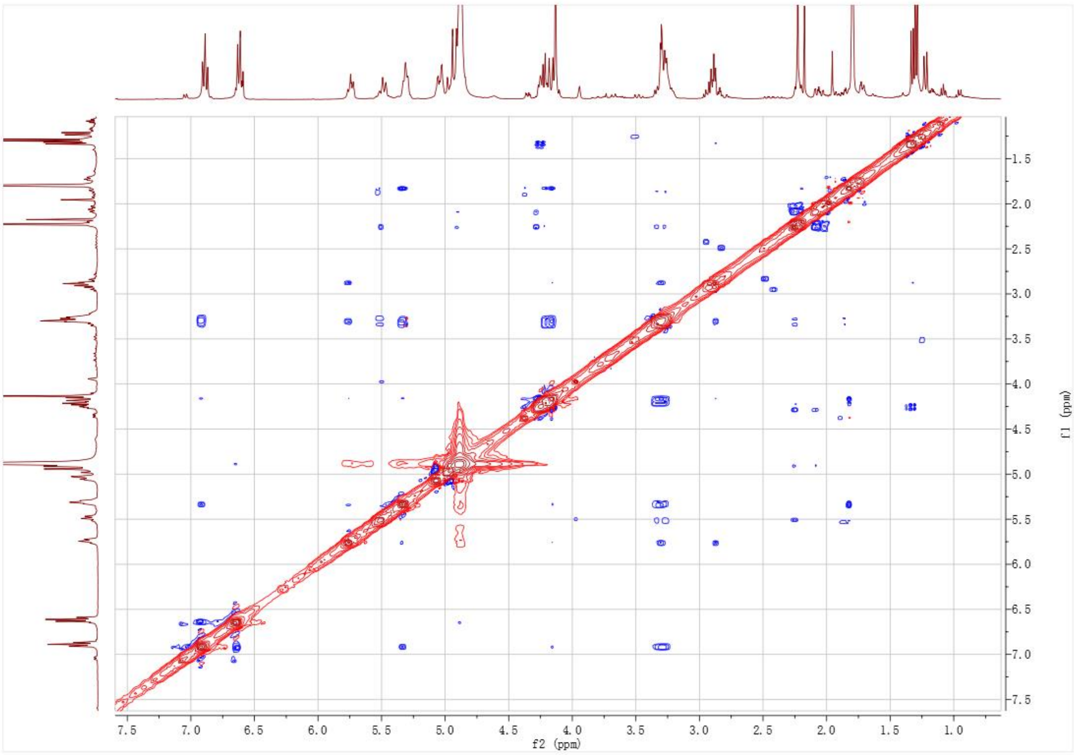

Figure S35 NOESY spectrum of the compounds **5/6** (CD_3OD)

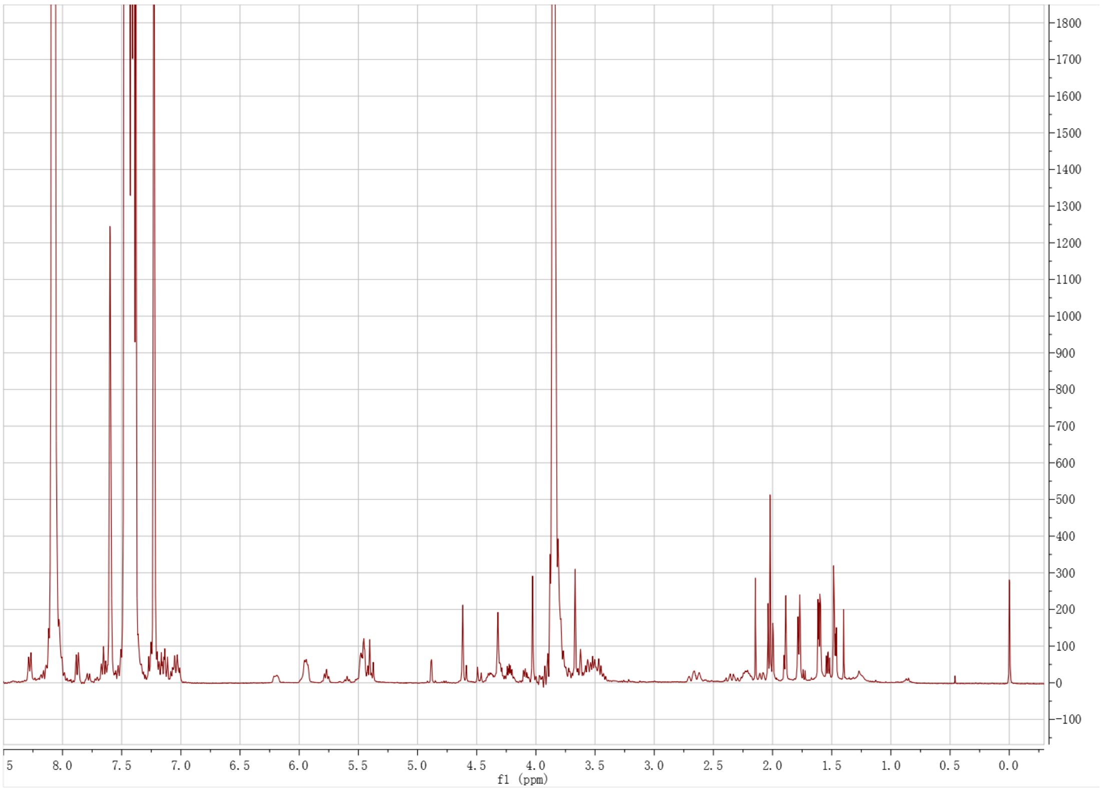

Figure S36 ^1H NMR spectrum of the compounds **2a** (400 MHz) (pyridine-d_6)

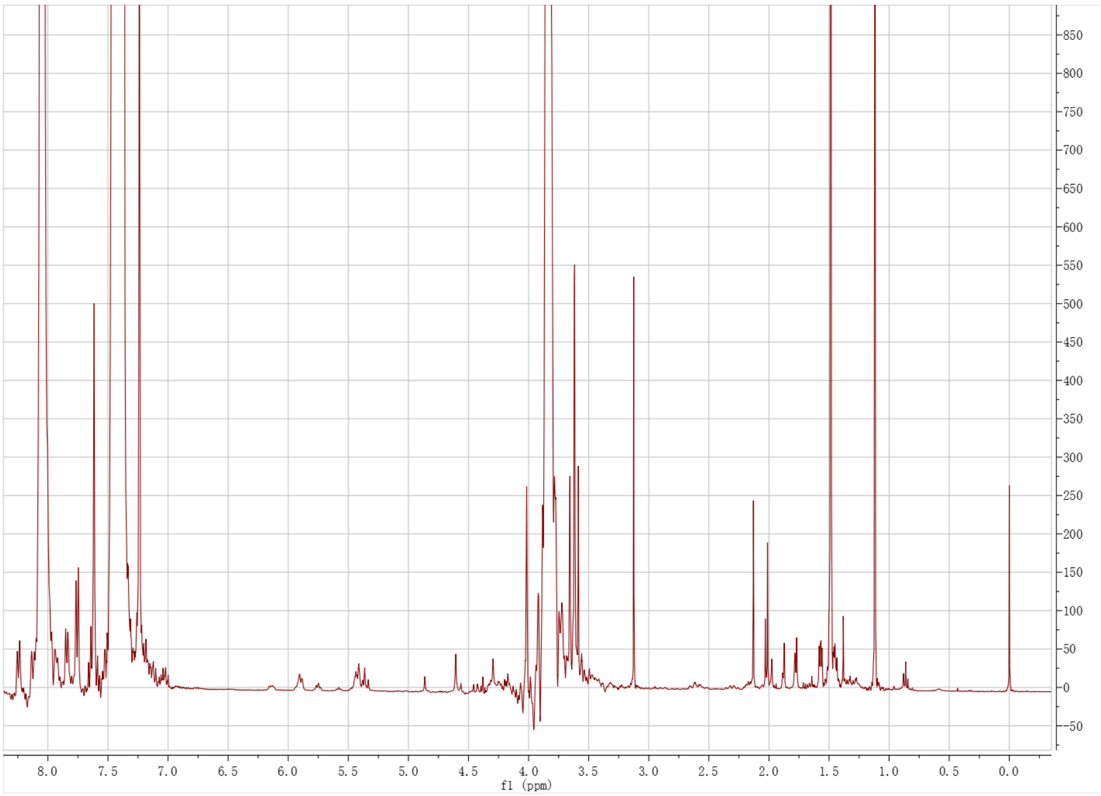

Figure S37 ^{1}H NMR spectrum of the compounds **2b** (400 MHz) (pyridine-d_6)

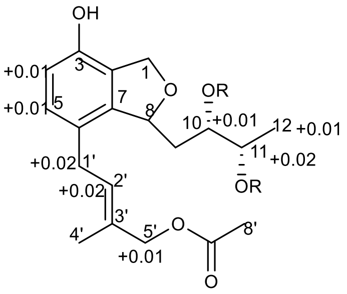

2a: R = (S)-MTPA
2b: R = (R)-MTRA

Figure S38. The Δδ (*δS–δR*) values from the (*S*)- and (*R*)-MTPA esters of 2.
